# NCOA4 linked to endothelial cell ferritinophagy and ferroptosis:a key regulator aggravate aortic endothelial inflammation and atherosclerosis

**DOI:** 10.1016/j.redox.2024.103465

**Published:** 2024-12-12

**Authors:** Li Zhu, Zijian Liu, Jiahui Liu, Zhenglong Li, Youli Bao, Xin Sun, Wenchen Zhao, An Zhou, Hongfei Wu

**Affiliations:** a School of Pharmacy, Anhui University of Chinese Medicine, Hefei, 230012, China; b Anhui Province Key Laboratory of Bioactive Natural Products; c Department of Pharmaceutical Sciences, School of Pharmacy, University of Pittsburgh, Pittsburgh, 15219, USA

**Keywords:** Nuclear receptor coactivator 4, Ferritinophagy, Ferroptosis, Endothelial injury, Atherosclerosis, “Gualou-Xiebai” herb pair

## Abstract

Atherosclerosis (AS) is associated with a high incidence of cardiovascular events, yet the mechanisms underlying this association remain unclear. Our previous study found that Atherosclerotic endothelial injury is closely associated with ferroptosis in ApoE^−/−^ mice. Ferroptosis is a novel mode of cell death induced by decreased antioxidant capacity of the organism and accumulation of reactive oxygen species. Nuclear receptor coactivator 4 (NCOA4)-mediated ferritinophagy is an important regulator of sudden ferroptosis in cells. However, the role of NCOA4 in AS and the exact mechanism by which it regulates the ferritinophagy response remain unclear. Herein, we report that NCOA4 expression is elevated in ApoE^−/−^ mice and endothelial cells and is significantly correlated with AS. NCOA4 expression promoted ferroptosis, and was positively correlated with ferritinophagy response. Mechanistically, our findings indicate that LOX-1 is a key upstream target that influences the function of NCOA4. The specific pathway is related to the activation of cGAS-STING signaling to upregulate NCOA4 expression. Moreover, our findings demonstrate the "Gualou-Xiebai" herb pair can regulate LOX-1 to inhibit ferroptosis. Collectively, our results provide evidence of a connection between NCOA4-mediated promotion of AS and suggest that targeting upstream molecules regulating NCOA4 could be a potential therapy for AS.

## 1 Introduction

Atherosclerosis (AS) is a vascular inflammatory disease characterized by the accumulation of lipids and inflammation of the arterial wall, which results from vascular endothelial injury, thickening of the arterial wall, and excessive secretion of mucus [1,2]. Vascular endothelial cells (ECs) injury and vascular inflammation is the AS the onset and progress of the crucial factors [3]. Injury to ECs facilitates the infiltration of circulating leukocytes into the vascular wall. This process triggers the releases of pro-inflammatory cytokines such as interleukin-1β (IL-1β) and tumor necrosis factor-α (TNF-α), along with an increase in cell adhesion molecule expression on ECs [4,5]. Concurrently, low-density lipoproteins (LDL) accumulate in the intima of blood vessels, leading to structural damage and the formation of fatty streaks in the vessel wall. These changes contribute to decreased elasticity and promote development of atherosclerotic plaques [6,7]. Therefore, inhibiting ECs is essential for advancing research into both prevention and strategies treatment of AS.

Ferroptosis is a form of cell death regulated by iron-dependent lipid peroxidation and the accumulation of reactive oxygen species (ROS) [8]. Recent studies have identified that ferroptosis as a pathogenic mechanism in the development of AS, highlighting its significant role in promoting lipid peroxidation, which leads to endothelial injury [9,10]. Consequently, targeting ferroptosis has emerged as a potential therapeutic strategy for AS. Given these insights, exploring the relationship between ferroptosis and AS-associated ECs injury may represent a breakthrough approach for treating AS.

Nuclear Receptor Coactivator 4 (NCOA4) serves as a selective cargo receptor for the autophagic degradation of ferritin within lysosomes [11]. NCOA4 binds to ferritin and facilitates its transport to autophagosomes. The subsequent fusion of autophagosomes with lysosomes results in the degradation of ferritin, thereby releasing Fe^2+^ into the cytoplasm; this process is referred to as ferritinophagy [12,13]. NCOA4 mediated ferritinophagy plays a crucial role in regulating ferroptosis in cells. However, aberrant activation of NCOA4 has been implicated in various pathological processes [14,15]. Oleic acid/palmitic acid -induced activation of ferritinophagy leads to iron accumulation and triggers ferroptotic responses in H9C2 cells, consequently promoting myocardial injury [16]. This suggests that NCOA4 may be involved in endothelial cell ferroptosis associated with AS through the mechanism of ferritinophagy. Nevertheless, it remains entirely unknown whether NCOA4 is activated and contributes to endothelial cell ferroptosis in HFD-induced AS mice.

In this study, we investigated the mechanistic link between NCOA4 and AS progression, while also exploring potential intervention mechanisms. This was achieved by constructing an AS model using ApoE^−/−^ mice and an oxidized low-density lipoprotein (ox-LDL)-induced injury model of human umbilical vein endothelial cells (HUVECs) in vitro. We evaluated the specific expression of NCOA4 in aortic plaques and injured endothelial cells in vitro and in vivo. Furthermore, we used tail vein injection of lentivirus in AS mice to establish overexpression and knockdown NCOA4 models to investigate the possible mechanistic link between ferritinophagy-induced ferroptosis and ECs injury. Additionally, we aimed to explore the regulatory mechanism of NCOA4 expression and assessed whether inhibition of the NCOA4 axis could slow down AS progression. Our findings suggest that targeting the NCOA4-mediated ferritinophagy pathway in endothelial cells may provide a novel strategy for preventing arterial endothelial injury.

## 2 Materials and methods

### 2.1 Materials

Gualou and Xiebai were purchased from Beijing Tong ren tang Co., Ltd. (Beijing, China; batch number: 211001, 211001). ox-LDL(YB001), 3-MA (HY19312; autophagy inhibitor), RU.521 (HY114180, cGAS-STING pathway inhibitor), Erastin was purchased from SparkJade (SJ-MX0039); Anti-NCOA4, Anti-GPX4, Anti-TFR1, Anti-FPN, Anti-cGAS, Anti-STING，Anti-ALOX15 were obtained from Immunoway (YT0302, YN3047, AF5343, YN8782, YT7062, YT5488, YT7564，Beijing). Anti-Ferritin，Anti-P62, Anti-LC3II/I, Anti-SR-A, Anti-CD36 were obtained from Abcam (ab75973, ab109012, ab192890, ab300632, ab252922, USA); Anti-ACSL4(DF12141), Anti-LOX-1(DF6522), Anti-SLC7A11 (26864-1AP), Anti-GAPDH (TA-08). Anti-VCAM-1, Anti-ICAM-1, (11444-1-AP, 60299-1-Ig), ELISA kit was obtained from Jianglai Biotechnology (Shanghai, China).

### 2.2 Animals

Male ApoE^−/−^ mice (20–22g, 6–8 weeks) and C57BL/6 J mice (20–22g, 6–8 weeks) were obtained from Jiangsu Hua Chuang Xin Nuo Pharmaceutical Technology Co. All animal experimental protocols received approval from the Ethics Committee of Anhui University of Traditional Chinese Medicine (approval number: AHUCM-mouse-2023011). Furthermore, all procedures for animal experiments were approved by the Ethical Review Committee for Laboratory Animal Welfare of Anhui University of Chinese Medicine.

### 2.3 Animal grouping and treatment

A model of AS was established by feeding ApoE^−/−^ mice a high-fat diet (40 % kcal fat, 20 % kcal protein, 40 % kcal carbohydrate, Cat #D12108C) for 12 weeks [16]. C57BL/6J mice of the same genetic background fed on regular chow were used as blank control group. ApoE^−/−^ mice were randomly divided into nine groups (n = 14): (a) high-fat diet (HFD) group; (b) HFD + NCOA4 overexpression group (LvNCOA4); (c) HFD + overexpression empty vector group (LvNC); (d) HFD + NCOA4 knockdown group (shNCOA4); (e) HFD + knockdown empty vector group (shNC); (f) HFD+(3 mg/kg/d) GLXB low dose group (GLXB-L); (g) HFD+(6 mg/kg/d) GLXB middle dose group (GLXB-M); (h) HFD+(12 mg/kg/d) GLXB high dose group (GLXB-H); (i) HFD + overexpression LOX-1+GLXB-H group (LvLOX-1+GLXB). Randomly selected ApoE^−/−^ mice with successful modelling, we used GLXB (3, 6, 12 g/kg/day) to continue the intervention in ApoE^−/−^ mice by gavage once daily for 4 weeks. Finally, serum and aorta were collected for further analysis.

### 2.4 GLXB extract preparation and treatment in vivo

The GLXB herbal pair consisted of two herbs (Gualou and Xiebai), which were purchased from Beijing Tong Ren Tang Co. Ltd (batch numbers: 211001,211001). The extracts were concentrated to a final concentration of 0.6 g/ml and stored at 4 °C in accordance with the methodology previously established by our research group [16].

Following the successful establishment of an HFD-induced AS model, daily gavage administration of either GLXB or saline was initiated for a duration of 4 weeks.

### 2.5 Lentivirus (LV)-infected ApoE^−/−^ mice

To overexpress NCOA4 and LOX-1 and knockdown NCOA4 in AS mice, LV18E-NCOA4, LV5-LOX-1 and NCOA4-Mus-1030/149/1345 mediated approaches were employed LV18E-NCOA4, LV5-LOX-1 and NCOA4-Mus-1030/149/1345 viruses were provided by Gemma Biologicals (Shanghai). Briefly, 80 μL of virus (titer: 1 × 10^9^ TU/ml) and 80 μL of empty vector (titer: 1 × 10^9^ TU/ml) were administered to the tail vein of ApoE^−/−^ mice. The genomic information was LV18E (CMV-MCS-hPGK-GFP-T2A-Puro), LV5 (EF-1a/GFP &Puro) LV3 (H1/GFP &Puro), Ncoa4-Mus-1030/149/1345.

### 2.6 HE staining

Paraffin sections of mouse aortic root tissue were prepared according to standard methods. The cut slices were placed in an oven at 60 °C for 30 min to dry. Dewaxing, hydration and haematoxylin and eosin (HE) staining were then carried out sequentially. The stained slices were added with drops of neutral gum, sealed with coverslips and placed under a microscope for observation and photography.

### 2.7 Transmission electron microscope (TEM) analysis

Fresh tissues or cells were fixed in 2.5 % glutaraldehyde for 24 h to preserve intracellular structures. They then underwent secondary fixation with osmium tetroxide for 2 h to improve contrast. Following this, samples were dehydrated and embedded to prepare ultrathin sections of approximately 70–90 nm thick. These sections were stained using a double staining method with lead citrate and uranyl acetate. Finally, the stained sections were examined using a transmission electron microscope.

### 2.8 Cell culture and treatment

The HUVECs were purchased from Bio-Channel (BC-C-HU-020). HUVECs were cultured in high-sugar medium (DMEM, Sparkjade, CA0002-500 ML), supplemented with 10 % serum fetal bovine (FBS, Gibco, 10099-141C) and 1 % penicillin-streptomycin at 37 °C, 5 % CO_2_.

To induce endothelial cell injury in vitro, HUVECs were treated with ox-LDL at a concentration of 100 μg/mL. Prior to the ox-LDL treatment, the cells received an addition of 3-MA at a concentration of 5 Mm [17]. Following transfection with NCOA4 overexpressing lentivirus for a duration of 24 h, the cells were subsequently exposed to RU.521 (10 μmol/mL) along with ox-LDL-containing medium for another period of 24 h [18]. The cells were then treated with RU.521 together with ox-LDL-containing medium.

### 2.9 Transfection

For plasmid transfection, HUVECs were inoculated in 6-well plates overnight and then transfected with the control plasmid using Lipo2000. After 6 h, the medium was replaced, and the cells were treated with ox-LDL for 24h post-transfection. HUVECs were also transfected with small interfering RNAs targeting NCOA4 (siNCOA4/siLOX-1) or lentivirus overexpressing NCOA4 of the specified sequences. The siRNAs used included siNCOA4-1, siNCOA4-2, siNCOA4-3, as well as siLOX-1-1, siLOX-1-2, and siLOX-1-3. Additionally, LV18E lentivirus (CMV-MCS-hPGK-GFP-T2A-Puro) was employed for transfection at a concentration of 5 μg/mL Polybrene.

### 2.10 Cell viability analysis

HUVECs were inoculated into 96-well plates (1 × 10^5^ cells/well) with 6 compound Wells in each group and treated with 100 μg/mL ox-LDL for 24h. After 10%CCK-8 solution was added and incubated for 1h away from light, the absorbance of each hole was quickly measured at 450 nm by enzyme-labeled instrument.

### 2.11 Enzyme-linked Immunosorbent assay (ELISA)

Aorta homogenate supernatant and cell culture supernatant were collected Operate according to the kit instructions, and test the absorbance value at 450 nm with an enzyme marker for calculation of the expression of TNF-α, IL-1β, ICAM-1, and VCAM-1. Cytokine concentrations were calculated using standard curves.

### 2.12 Western blot analysis

Total proteins were extracted from aortic tissue and HUVECs by polyacrylamide gel electrophoresis, and protein lysates were transferred to PVDF transfer membranes, which were then sealed and exposed to appropriate antibodies. Protein bands were detected using an ECL chemiluminescence kit (Thermo Fisher Scientific) and analyzed using Image J software.

### 2.13 Immunofluorescence analysis (IF)

HUVECs suspensions were seeded in 6-well plates. Following the appropriate drug treatment, formaldehyde was added and the cells were fixed at room temperature for 15min. The cells were permeabilized with Triton X-100 for 10 min, followed by addition of fluorescence-blocking solution (5%BSA) and standing at room temperature for 1 h. Then the cells were incubated with primary antibody at 4 °C overnight and incubated with secondary antibody at room temperature for 1 h. The nuclei were then stained with DAPI and stood at room temperature for 5 min. Finally, the cells were photographed using a fluorescence microscope.

### 2.14 Quantitative Real-time polymerase chain reaction (qRT-PCR)

RNA was isolated using a total RNA (1 μg) extraction kit. According to the TaKaRa reverse transcription kit, the total RNA is reversely transcribed into cDNA. The mRNA levels of target genes in HUVECs were detected according to TaKaRa fluorescence quantitative (TB Green) kit instructions. The 2^-△△CT^ values of each sample were calculated, and the differences among the groups were analyzed by SPSS software.

### 2.15 Flow cytometry analysis

HUVECs suspension was inoculated into 6-well plates, and different treatments were performed among each group. The prepared FerroOrange (Fe^2+^ probe) working liquid of 1 μmol/L was added, mixed and incubated in the cell incubator for 30 min under dark light. PBS was resuspended 5 times, analyzed by Flow cytometry and quantified by Flow Jo.

### 2.16 Analysis of GSH, SOD, MDA and Fe content

The content of GSH/SOD/MDA/Fe was determined according to the instructions of the biochemical kit Nanjing, China.

### 2.17 C11-BODIPY581/591 probe analysis

C11-BODIPY581/591 can be used to detect intracellular lipid peroxidation. HUVECs were inoculated into 6-well plates at a density of 2.5 × 10^5^ cells per well, After different treatment among the groups. Next, the cells were collected and analyzed via flow cytometry using the C11-BODIPY581/591 lipid ROS detection kit instructions.

### 2.18 Co-immunoprecipitation experiment

Cell extracts were incubated overnight at 4 °C with specific antibodies in extraction buffer to form immune complexes. Protein A-agarose beads were then added and incubated for an additional 2 h at 4 °C to precipitate the complexes. The precipitates were washed five times with extraction buffer to eliminate non-specific bindings. After washing, the pellets were eluted using SDS sample buffer and heated at 95 °C for denaturation prior to SDS-PAGE electrophoresis.

### 2.19 Statistical analysis

Data were analyzed using SPSS version 22.0 and GraphPad Prism version 8.0. Data are expressed as mean ± SD. One-way analysis of variance (ANOVA) or two-way ANOVA was employed for the comparisons among multiple groups, following Tukey's multiple comparisons test, and the Dunnetts test was used when the variance was not uniform. *P* < 0.05 is considered statistically significant.

## 3 Results

### 3.1 NCOA4 is upregulated in atherosclerotic mice

**Fig. 1:**
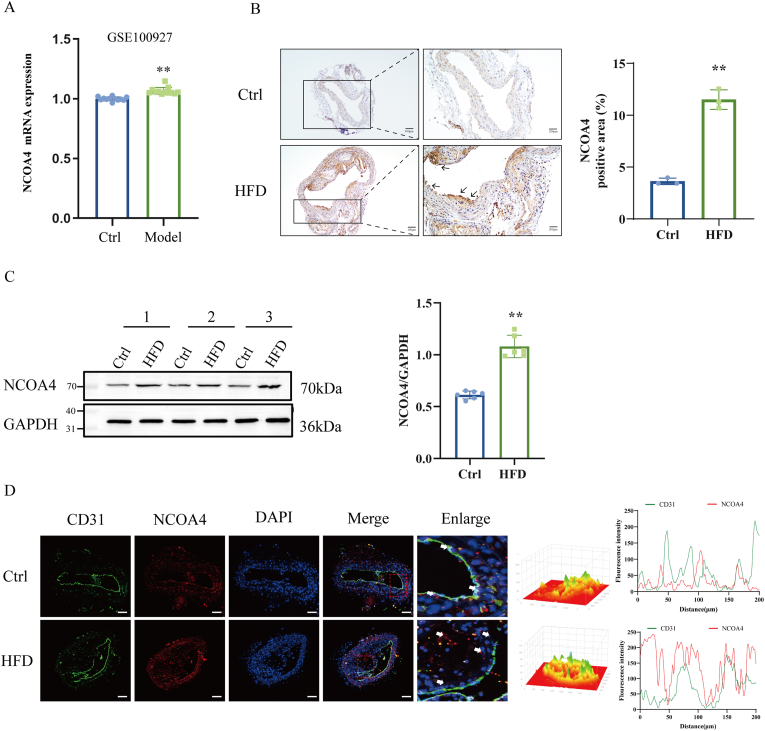
NCOA4 expression is upregulated in atherosclerotic mice. (A) Analysis of NCOA4 expression in GSE100927 data (n = 12). (B) Representative images of immunohistochemical staining of NCOA4 in the aorta of ApoE^−/−^ mice following 12 weeks of HFD. NCOA4 increased in the aortic arch (scale bar: 50 μm, n = 3). (C) Western blotting analysis of NCOA4 in the aortic (n = 6). (D) Immunofluorescence staining of CD31 (green), NCOA4 (red), and DAPI (blue) in mouse aorta (scale bar: 200 μm, n = 3). ∗*P* < 0.05, ∗∗*P* < 0.01 vs. control group.

To clarify the relationship between NCOA4 and AS lesion formation, we analyzed RNA-seq data from human atherosclerotic plaque obtained from the National Center for Biotechnology Information Gene Expression Omnibus (GEO) databases (GSE100927). As shown in Fig. 1A, the messenger RNA (mRNA) expression level of NCOA4 was upregulated in atherosclerotic plaques. To further support the potential role of NCOA4 in AS, we established an AS model by feeding ApoE^−/−^ mice with a high-fat diet for 12 weeks. Doppler ultrasonography revealed lipid deposition within the aorta, and plaque area was quantified through haematoxylin-eosin (HE) staining to confirm successful model establishment (Supplementary Figs. S1A–C). Immunohistochemical analysis demonstrated an increased expression level of NCOA4 within the endothelial cells of AS mice (Fig. 1B). To investigate how AS progression influences NCOA4 expression, we assessed both protein levels and nuclear localization of NCOA4 in mouse aortas. In HFD-fed mice, levels of NCOA4 protein were higher than those in control group mice (Fig. 1C). Furthermore, while NCOA4 predominantly localized to the nucleus under normal conditions, it was primarily distributed within the cytoplasm in HFD group (Fig. 1D). These findings suggest that endothelial cell-associated NCOA4 may be positively correlated with the progression of AS.

### 3.2 NCOA4 regulates the development of atherosclerotic lesions in HFD-induced ApoE^−/−^ mice

**Fig. 2:**
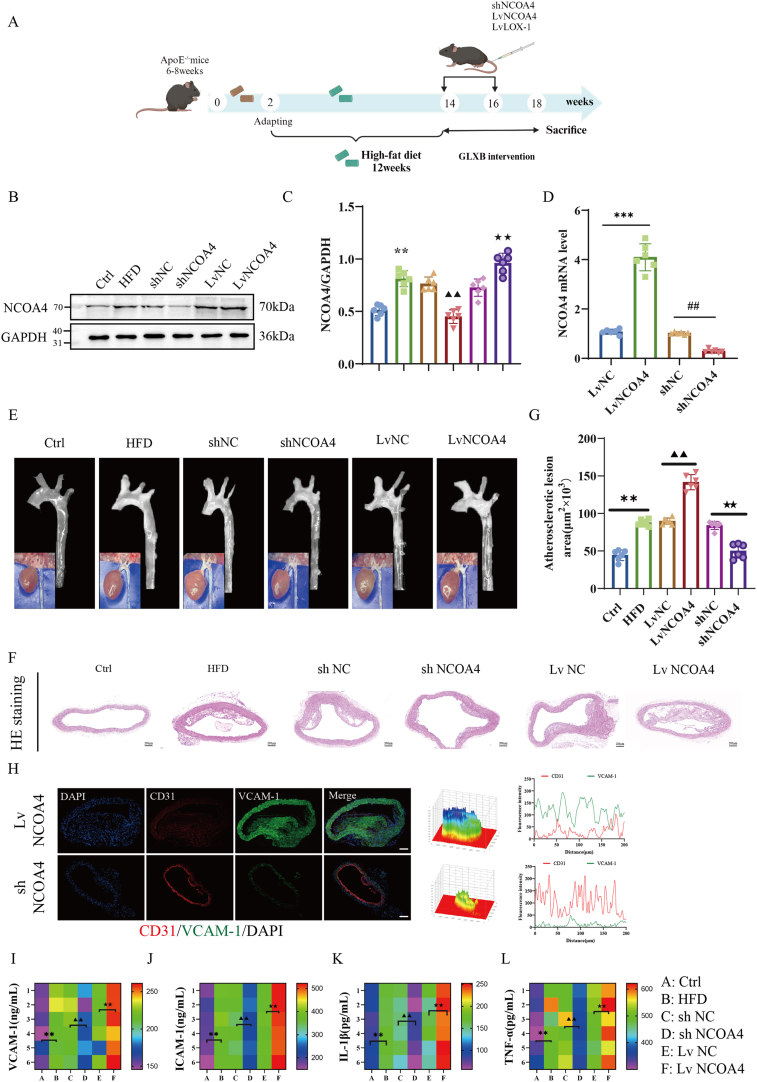
NCOA4 regulates the development of atherosclerotic lesions in HFD-induced ApoE^−/−^ mice. (A) Experimental schedule of AS mice model and stable model with tail vein injection of NCOA4. (B–D) The validation of NCOA4 lentiviral transfection efficiency (n = 6). (E) Aortic imaging showing aortic lipid deposition in each group. (F–G) HE staining of aortic in each group (scale bar: 200 μm, n = 6). (H) Immunofluorescence staining of CD31 (green), VCAM-1 (red), and DAPI (blue) in mouse aorta (scale bar: 200 μm, n = 6). (I–L) Measurement of VCAM-1, ICAM-1, TNF-α, and IL-1β levels were determined within the aorta using commercially respective kits (n = 6). ∗*P* < 0.05, ∗∗*P* < 0.01 vs. control group; ^▲^*P* < 0.05, ^▲▲^*P* < 0.01 vs. shNC group; ^★^*P* < 0.05, ^★★^*P* < 0.01 vs. LvNC group.

To investigate the role of NCOA4 in high-fat diet (HFD)-induced AS, we established stable models of NCOA4 overexpression and knockdown via tail vein injection (Fig. 2A). The deletion and overexpression of the NCOA4 in mouse aortas were validated at both mRNA and protein levels (Fig. 2B–D). Furthermore, immunofluorescence staining for CD31 (green), NCOA4 (red), and DAPI (blue) provided additional confirmation of these findings (Supplementary Fig. S2). Imaging and HE staining results from mouse aortas indicated that compared to the NCOA4 overexpression group, knockdown of NCOA4 significantly reduced plaque area and lipid deposition at the aortic arch. Conversely, overexpression of NCOA4 resulted in a substantial increase in plaque area and lipid accumulation within that region (Fig. 2E–G).

Endothelial cells play a crucial role in regulating intravascular homeostasis, and injured cells release various physicochemical signals (such as VCAM-1, ICAM-1, TNF-α, and IL-1β) on their surface, leading to increased vascular permeability and the induction of vascular inflammation [19,20]. To explore whether AS is mediated by endothelial cell-specific NCOA4 expression, we observed that VCAM-1 fluorescence expression was significantly lower in the arterial endothelium of mice with knocked-out NCOA4 compared to those with its overexpression (Fig. 2H). Furthermore, plasma levels of VCAM-1, ICAM-1, TNF-α, and IL-1β were also notably decreased in these mice (Fig. 2I–L). Overall, these data suggest that loss of endothelial cell-specific NCOA4 effectively mitigates the progression of atherosclerotic lesions.

### 3.3 NCOA4 silence attenuates ox-LDL-induced HUVECs injury

**Fig. 3:**
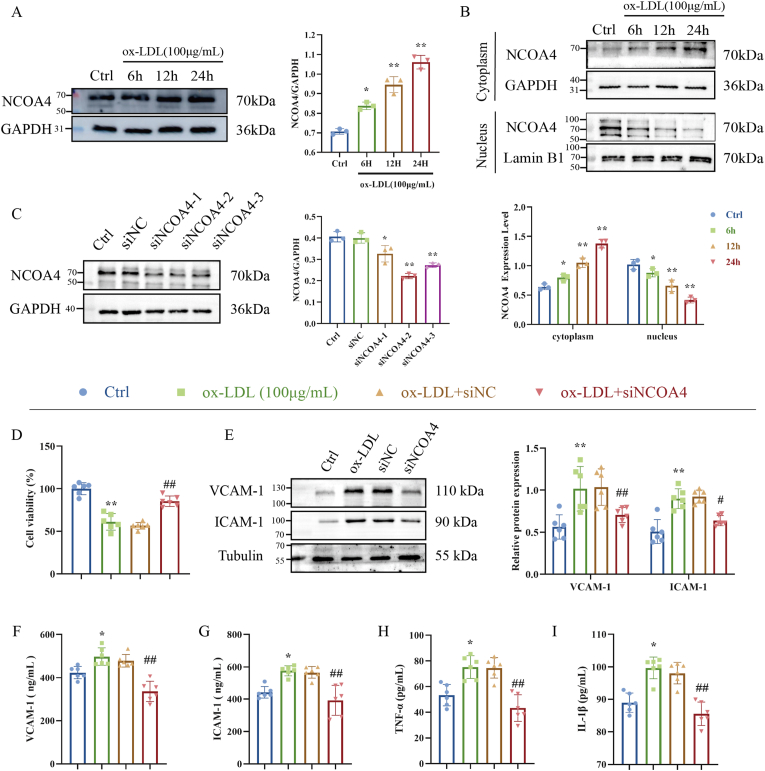
NCOA4 silence inhibits ox-LDL-induced inflammatory injury in HUVECs. (A) Western blot to detect NCOA4 expression in injured HUVECs (n = 3). (B) Western blot to detect NCOA4 expression in cytoplasm and nucleus (n = 6). (C) Transfection efficiency of NCOA4 siRNA (n = 3). (D) CCK-8 to detect the effect of silencing NCOA4 on cell viability (n = 6). (E) Western blot to detect ICAM-1 and VCAM-1 in injured HUVECs (n = 6). (F–I) ELISA kits to detect the effect of silencing NCOA4 on the levels of inflammatory factors (n = 6). ∗*P* < 0.05, ∗∗*P* < 0.01 vs. control group, ^#^*P* < 0.05, ^##^*P* < 0.01 vs. siNC group.

To investigate the role of NCOA4 in endothelial cell injury, we utilized ox-LDL to simulate endothelial injury caused by lipid accumulation in AS. As shown in Fig. 3A, the expression level of NCOA4 in HUVECs increased in a dose-dependent manner following exposure to ox-LDL (from 6h to 24h). We then examined the effects of ox-LDL on the cytoplasmic and nuclear levels of NCOA4. Notably, treatment with ox-LDL significantly inhibited nuclear protein levels of NCOA4 while promoting its elevation within the cytoplasm (Fig. 3B). Subsequently, we assessed the biological implications of NCOA4 by constructing stable NCOA4-silenced HUVECs. Western blot analysis confirmed the effectiveness of NCOA4 silencing (Fig. 3C).

Interestingly, our findings revealed that silencing NCOA4 alleviated viability loss induced by ox-LDL in HUVECs (Fig. 3D). Furthermore, NCOA4 silencing reduced the protein expression levels of adhesion molecules ICAM-1 and VCAM-1 induced by ox-LDL exposure in HUVECs (Fig. 3E). Similar results were observed when analyzing conditioned media collected from injured HUVECs cultures; specifically, siRNA-mediated knockdown of NCOA4 led to significant reductions in inflammatory factors including ICAM-1, VCAM-1, TNF-α, and IL-1β (Fig. 3F–I). In summary, silencing NCOA4 enhances cell viability and suppresses the release of inflammatory factors in injured HUVECs.

### 3.4 NCOA4 knockdown inhibits ferroptosis and ferritinophagy of atherosclerotic endothelial cells

**Fig. 4:**
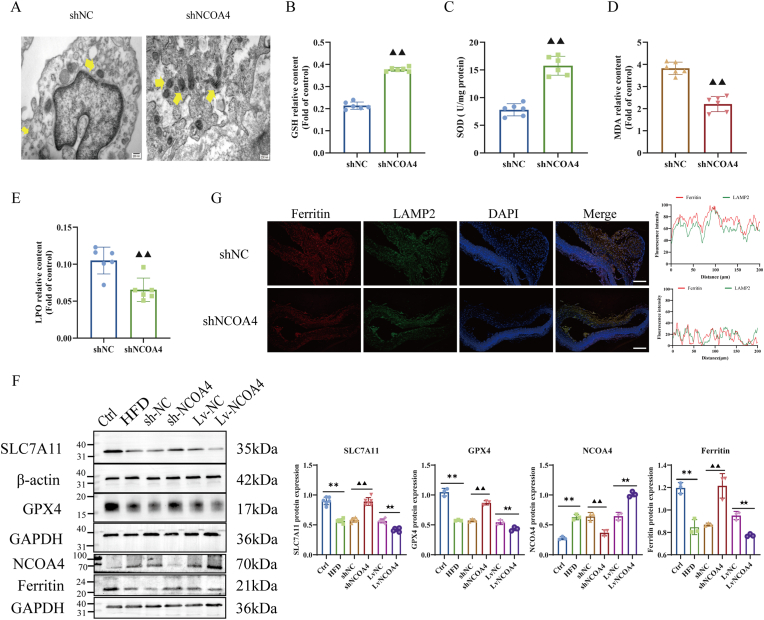
NCOA4 knockdown inhibits ferroptosis and ferritinophagy of atherosclerotic endothelial cells. (A) TEM to detect the mitochondrial injury in the aortic endothelial cells of AS mice (n = 6). (B–E) Biochemical kits to detect the expression of lipid peroxidation levels of MDA, LPO, GSH, and SOD in the aortic tissues of AS mice (n = 6). (F) Immunofluorescence staining of Ferritin (red) LAMP2 (green) are co-localized in sections of atherosclerotic lesions (n = 6). (G) Western blot to detect the expression of ferroptosis proteins GPX4 and SLC7A11 and the level of ferritinophagy protein in the aorta of AS mice (n = 6). Scale bar, 100 μm ∗*P* < 0.05, ∗∗*P* < 0.01 vs. control group; ^▲^*P* < 0.05, ^▲▲^*P* < 0.01 vs. shNC group; ^★^*P* < 0.05, ^★★^*P* < 0.01 vs. LvNC group.

To investigate the specific mechanisms underlying NCOA4-induced endothelial cell injury, we first assessed the extent of endothelial cell damage using transmission electron microscopy (TEM). As shown in Fig. 4A, knockdown of NCOA4 significantly alleviated mitochondrial damage in aortic endothelial cells. In contrast, overexpression of NCOA4 exacerbated mitochondrial swelling and disrupted the structure of mitochondrial cristae—morphological changes characteristic of ferroptosis (Supplementary Fig. S4A). Excessive iron accumulation leads to the generation of toxic lipid peroxides via Fenton reactions, which play a crucial role in ferroptosis [9]. Prussian blue staining results indicated that silencing NCOA4 markedly inhibited iron deposition in the aorta compared to control groups with empty vectors. Conversely, significant increases in iron deposition were observed in the NCOA4 overexpression group (Supplementary Fig. S3).

Consistent with these findings, we discovered that mice lacking NCOA4 exhibited reduced levels of lipid peroxides (LPO) and malondialdehyde (MDA), while levels of antioxidants such as glutathione (GSH) and superoxide dismutase (SOD) were significantly elevated when compared to mice with knockout empty vectors (Fig. 4B–E). This phenomenon sharply contrasted with observations from NCOA4 overexpression groups (Supplementary Figs. S4B–E). Furthermore, western blotting analysis revealed that knockdown of NCOA4 enhanced expression levels of anti-ferroptotic markers including glutathione peroxidase 4 (GPX4) and solute carrier family 7 member 11 (SLC7A11) within the aorta. Conversely, overexpression of NCOA4 significantly suppressed GPX4 and SLC7A11 protein levels (Fig. 4F). Collectively, these findings suggest that NCOA4 serves as an important factor promoting ferroptosis in aortic endothelial cells during AS. However, further elucidation is required regarding the potential mechanisms by which NCOA4 regulates ferroptosis within endothelial cells affected by AS.

To further explore how HFD-induced conditions affect ferritinophagy mediated by NCOA4 in arterial endothelial cells, we found that overexpression of NCOA4 significantly reduced ferritin levels as illustrated in Fig. 4F; meanwhile, knockdown NCOA4 resulted in increased ferritin levels without confirming whether ferritinophagy occurred. Subsequently, we evaluated its autophagic degradation status through co-localization studies between transport proteins and LAMP2 under immunofluorescence conditions (Fig. 4G, Supplementary Fig. S4F). In summary, our findings indicate that in atherosclerotic mice, knockdown of NCOA4 inhibits both ferroptosis and ferritinophagy.

### 3.5 NCOA4 aggravates ox-LDL-induced ferroptosis in HUVECs

**Fig. 5:**
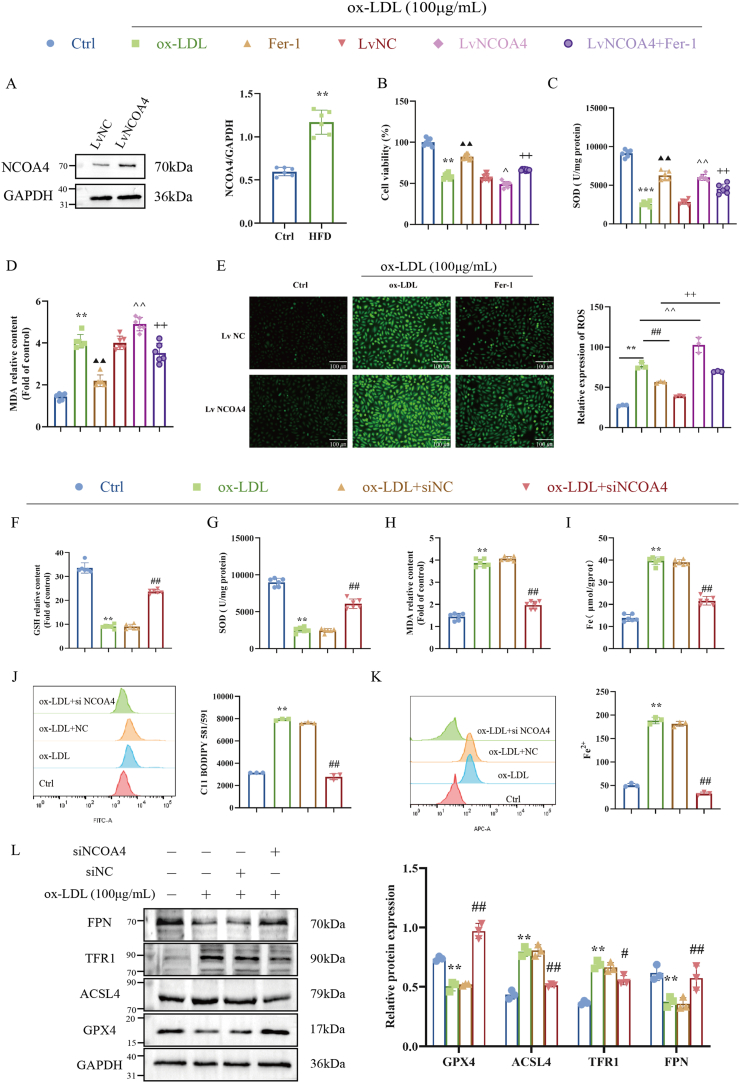
NCOA4 silence inhibits ox-LDL-induced ferroptosis in HUVECs. (A) Transfect HUVECs with a designated lentivirus carrying the NCOA4 gene to achieve stable overexpression with an empty vector. And, then harvested for immunoblot analysis. Cells were treated with the indicated dose of ox-LDL with or without 1 μmol/L Fer-1 and then the cell viability (B), SOD MDA levels (C–D), and ROS levels (E) were analyzed. (F–I) Biochemical kit for GSH, SOD MDA and Fe levels (n = 6). (J) C11-BODIPY581/591 for lipid ROS levels (n = 3). (K) Flow cytometry for Fe^2+^ levels (n = 3). (L) Western blot to detect the expression levels of GPX4, ACSL4, TFR1 and FPN proteins in HUVECs treated with ox-LDL for 24h and NCOA4 siRNA (siNCOA4) for 24 h before ox-LDL exposure (n = 3). ∗*P* < 0.05, ∗∗*P* < 0.01 vs. control group, ^#^*P* < 0.05, ^##^*P* < 0.01 vs. siNC group.

The elevation of ferroptosis levels plays a pivotal role in precipitating endothelial cell injury; however, the relationship between this phenomenon and NCOA4 activation remains inadequately explored. Consequently, we investigated the effects of NCOA4 overexpression on ferroptosis levels and cell viability in HUVECs. NCOA4 overexpression model (LvNCOA4) was established using lentiviral vectors (Fig. 5A). Our findings demonstrated that MDA production levels and cytotoxicity were significantly increased, while SOD levels are notably decreased in the LvNCOA4 group of HUVECs (Fig. 5B–D). Moreover, under stimulated conditions, HUVECs from the LvNCOA4 group exhibited elevated rates of cell death alongside heightened reactive oxygen species (ROS) levels. Notably, these alterations could be mitigated by treatment with the ferroptosis inhibitor Fer-1 (Fig. 5E). Subsequently, we used JC-1 staining to assess mitochondrial membrane potential changes after Fer-1 treatment in cells from the LvNCOA4 group (Supplementary Fig. S5A). The results indicated that JC-1 accumulated as red fluorescence within normal HUVECs mitochondria; however, exposure to ox-LDL (100 μg/mL) resulted in dissipation of ΔΨm and an increase in green fluorescence associated with monomeric JC-1. Furthermore, combined treatment with Fer-1 exacerbated ΔΨm dissipation induced by cells from the LvNCO4 group. These data suggest that NCOA4 activation directly contributes to ferroptosis induction.

Next, we investigated the levels of GSH and SOD, as well as the oxidative stress marker MDA and observed that NCOA4 silence significantly increased the levels of GSH and SOD while reducing the MDA content in HUVECs (Fig. 5F–H). The accumulation of lipid reactive oxygen species (ROS) was assessed using the C11-BODIPY (581/591) probe. As shown in Fig. 5J, lipid peroxidation levels were notably elevated in injured HUVECs but decreased after NCOA4 silencing. Moreover, we also examined total iron and Fe^2+^ levels, which were significantly reduced by NCOA4 silence in HUVEC (Fig. 5I–K). Western blot analysis results showed that NCOA4 silence significantly reversed the ox-LDL-induced time-dependent reduction of GPX4 and FPN protein expression and significantly down-regulated the protein expression of ACSL4 and TFR1 (Fig. 5L, Supplementary Fig. S5B). Taken together, these findings strongly suggested that NCOA4 silence can inhibit ferroptosis in HUVECs, which was consistent with the in vivo results.

### 3.6 NCOA4-mediates ferritinophagy promotes ox-LDL-induced HUVECs ferroptosis

**Fig. 6:**
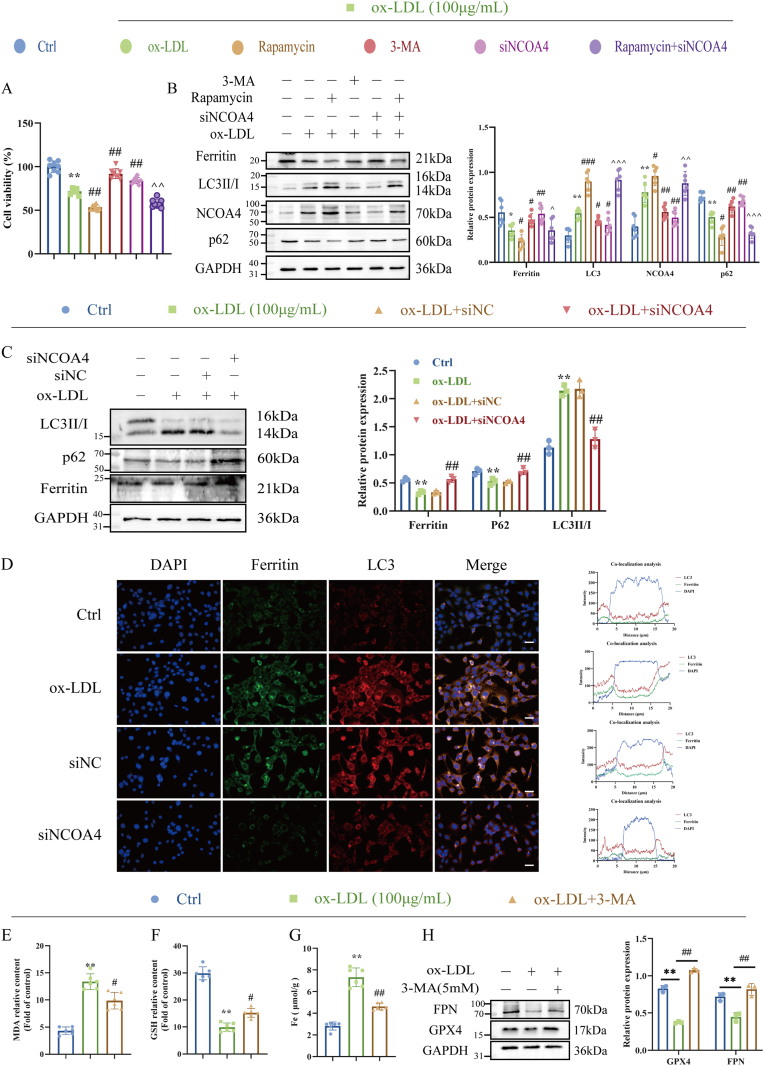
NCOA4-mediates ferritinophagy promotes ox-LDL-induced HUVECs ferroptosis. (A) CCK-8 to detect the cell viability (n = 6). (B)Treatment of HUVECs with 100 μg/mL ox-LDL for 24h and specific Rapamycin (100 nm) for 24h before ox-LDL exposure. Cells were then harvested for western blot analysis (n = 6). (C) Western blot detected the levels of ferritinophagy-associated protein LC3, P62, NCOA4 and Ferritin. (D) Immunostaining of ferritin and LC3 in HUVECs (DAPI, blue; LC3, red; ferritin, green). Scale bar: 100 μm (n = 3). (E–G) The level of MDA, GSH and Fe in HUVECs (n = 6). (H) Western bolt analysis of GPX4 and FPN expression in HUVECs treated with 3-MA (5 mM) 1h then treated with ox-LDL for 24 h (n = 3). ∗*P* < 0.05, ∗∗*P* < 0.01 vs. control group; ^#^*P* < 0.05, ^##^*P* < 0.01 vs. ox-LDL group; ^ʌ^*P* < 0.05, ^ʌʌ^*P* < 0.01 vs. Rapamycin group.

It is noteworthy that autophagy plays a complex role in regulating the activation of NCOA4 [19,20]. To investigate whether autophagy promotes the activation of NCOA4, we treated HUVECs with the autophagy inducer Rapamycin and either transfected or did not transfect NCOA4. The results indicated that after stimulation with ox-LDL or treatment with Rapamycin, HUVECs transfected with the NCOA4 plasmid exhibited a significant decrease in both cell viability (Fig. 6A) and ferritin levels (Fig. 6B). This suggests that the downregulation of ferritin is attributable to autophagy-dependent degradation. Importantly, inhibition of autophagy was found to significantly restore ferritin expression levels (Fig. 6B). Therefore, the addition of rapamycin further corroborated that NCOA4-mediated ferritinophagy indeed relies on the process of autophagy itself; but, inhibiting autophagy slows down ferritinophagy.

Next, we investigated the role between NCOA4 silence and ferritinophagy by Western blot. We found that ox-LDL upregulated the protein levels of LC3II (an autophagy marker) in a time-dependent manner (Supplementary Fig. S6), indicating the presence of autophagy activation. NCOA4 silence significantly reversed the elevated LC3 levels (Fig. 6C). Immunofluorescence analysis showed that NCOA4 silence reduced the co-localization of Ferritin and LC3 (Fig. 6D). To clarify whether ferritinophagy is the triggering mechanism of NCOA4 regulation of ferroptosis in HUVECs, the classical autophagy inhibitor 3-methyladenine (3-MA) was used to inhibit the level of ferritinophagy in HUVECs (Supplementary Fig. S7B). Pretreatment with 3-MA attenuated ferroptosis, as evidenced by increased cell viability (Supplementary Fig. S7A) and reduced MDA production (Fig. 6E), GSH depletion (Fig. 6F), decreased iron overload (Fig. 6G) as well as increased the expression of FPN and GPX4 (Fig. 6H). These data suggest that ferroptosis induced by ox-LDL requires activation of ferritinophagy.

### 3.7 LOX-1 is involved in ox-LDL-induced HUVECs injury and AS

**Fig. 7:**
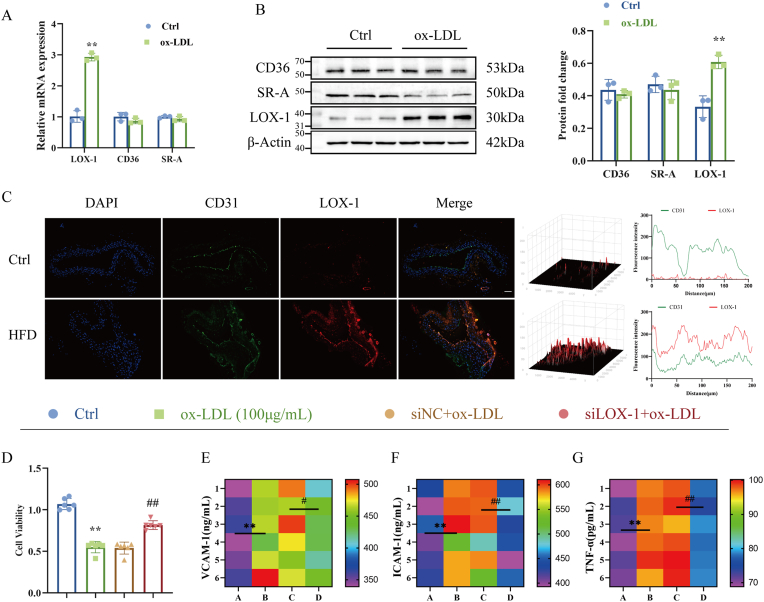
LOX-1 accelerates ox-LDL-induced inflammatory injury in HUVECs. (A–B) CD36, LOX-1 and SR-A mRNA and protein levels were detected by RCR and Western blot in ox-LDL-treated HUVECs for 24h (n = 3). (C) Immunofluorescence staining of CD31 (green) LOX-1 (red) are co-localized in sections of atherosclerotic lesions. Scale bar: 100 μm (n = 3). (D) Cell viability of HUVECs following ox-LDL treatment for 24 h (n = 6). (E–G) ELISA kit to detect the effect of silencing LOX-1 on the levels of inflammatory factors ICAM-1, VCAM-1, and TNF-α (n = 6). ∗*P* < 0.05, ∗∗*P* < 0.01 vs. control group, ^#^*P* < 0.05, ^##^*P* < 0.01 vs. siNC group.

Vascular endothelial injury often derives from dysregulation of intracellular lipid metabolism due to uncontrolled uptake of lipoproteins [[21], [22], [23]]. Lipoprotein uptake and clearance are primarily controlled by the scavenger receptor family proteins SR-A, CD36 and LOX-1 [24,25]. To investigate the key scavenger receptor involved in endothelial cell injury, we used PCR and western blot to screen for scavenger receptor expression. As shown in Fig. 7A, the expression levels of LOX-1 mRNA and protein were significantly increased, whereas the expression levels of CD36 and SR-A were not statistically different (Fig. 7B). Furthermore, co-localization levels of CD31 and LOX-1 also increased significantly in the aortas of ApoE^−/−^ mice, indicating that LOX-1 is involved in endothelial cell injury in AS (Fig. 7C).

Next, we evaluated the biological effects of LOX-1 by generating stable LOX-1-silence HUVECs and verified the silencing efficiency of LOX-1 by Western blot (Supplementary Fig. S8). Interestingly, we found that LOX-1 silence significantly suppressed ox-LDL-induced loss of HUVECs viability (Fig. 7D). Furthermore, the increase in the levels of ICAM-1, VCAM-1 and TNF-α in the ox-LDL - treated group was significantly reversed with LOX-1 silence (Fig. 7E–G). These results indicated that LOX-1 silence decreased ox-LDL-induced inflammatory injury in HUVECs.

### 3.8 LOX-1 silence inhibits ferroptosis in ox-LDL-induced HUVECs

**Fig. 8:**
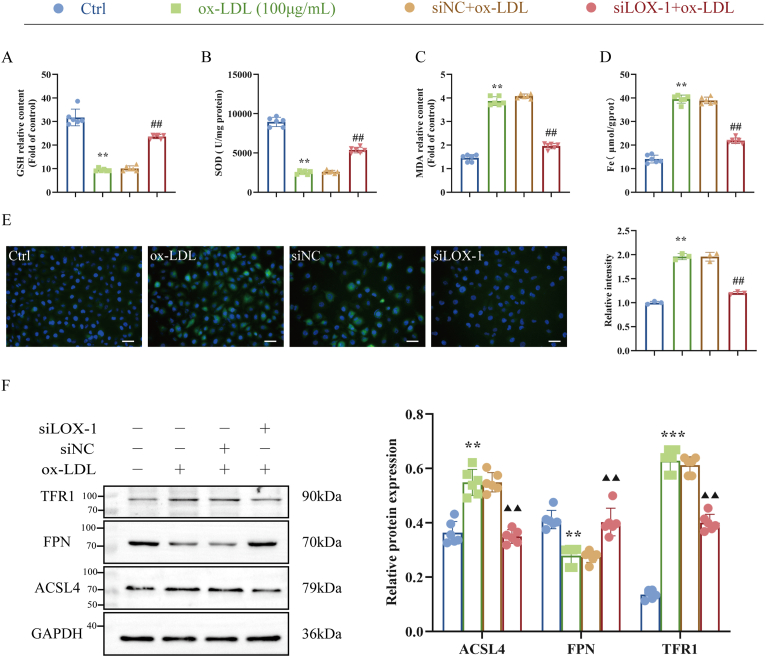
LOX-1 silence inhibits ox-LDL-induced ferroptosis in HUVECs. (A–C) Biochemical kit to detect lipid peroxidation level (n = 6). (D) Iron kit to detect total intracellular iron content (n = 6). (E) Immunofluorescence to detect intracellular ROS level (n = 3). (F) Western blot to detect the expression of ferroptosis marker proteins TFR1, ACSL4 and FPN (n = 6). ∗*P* < 0.05, ∗∗*P* < 0.01 vs. control group, ^#^*P* < 0.05, ^##^*P* < 0.01 vs. siNC group.

To determine whether LOX-1 promotion of HUVECs injury was related to ferroptosis, we examined lipid peroxidation levels and ferroptosis regulated proteins in HUVECs. Our results indicated that LOX-1 silence reversed the ox-LDL-induced SOD and GSH levels and inhibited the increase in MDA and total iron levels in HUVECs compared with the ox-LDL group (Fig. 8A–D). The ROS fluorescence results further validated above results, confirming that LOX-1 silencing significantly suppressed lipid peroxide accumulation in injured HUVECs (Fig. 8E). In addition, LOX-1 silence reversed the ox-LDL-induced increase in the expression of TFR1 and ACSL4 in HUVECs, and significantly increased the expression of FPN in HUVECs compared with ox-LDL group (Fig. 8F). These results suggest that LOX-1 silence inhibits ferroptosis levels in injured HUVECs.

### 3.9 cGAS-STING signaling pathway was activated by LOX-1 and promoted NCOA4-mediated HUVECs ferroptosis

**Fig. 9:**
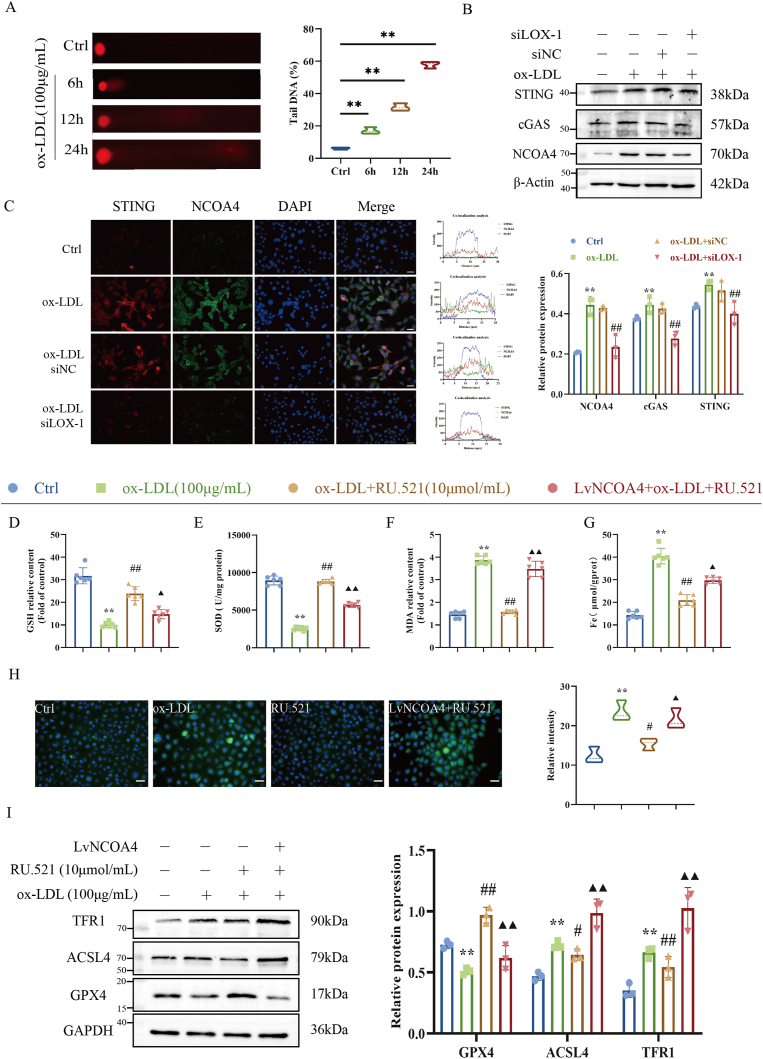
cGAS-STING signaling pathway activated by LOX-1 promotes NCOA4-mediated HUVECs ferroptosis. (A) Comet assay to detect cellular DNA damage (n = 3). (B) Western blot to detect cGAS, STING and NCOA4 proteins (n = 3). (C) Immunofluorescence co-localization to observe the relationship between STING and NCOA4 (DAPI, blue; STING, red; NCOA4, green). Scale bar: 100 μm. (D–F) Biochemical kit to detect lipid peroxidation level (n = 6). (G) Iron kit to detect total intracellular iron content (n = 6). (H) Immunofluorescence to detect intracellular ROS level (n = 3). (I) Western blot to detect the expression of ferroptosis proteins TFR1, ACSL4 and FPN (n = 3). ∗*P* < 0.05, ∗∗*P* < 0.01 vs. control group; ^#^*P* < 0.05, ^##^*P* < 0.01 vs. ox-LDL group; ^▲^*P* < 0.05, ^▲▲^*P* < 0.01 vs. ox-LDL + RU.521 group.

The cGAS-STING signaling pathway is an important DNA sensing mechanism mediating the innate immune response and has emerged as a key mediator of inflammation, playing a critical role in the ferroptosis [18,26]. Comet assay results showed that ox-LDL enhanced the tailing phenomenon of HUVECs, and the degree of tailing increased time-dependently (Fig. 9A), and the expression of cGAS-STING proteins increased, suggesting that ox-LDL is able to trigger DNA damage and activate intracellular DNA sensing mechanism pathway proteins in the process of ox-LDL-induced HUVECs ferroptosis. The cGAS-STING pathway has been shown to be closely related to autophagy, and inhibiting the cGAS-STING pathway can significantly inhibit ferritinophagy [27,28]. As shown in Fig. 9B, LOX-1 silence could significantly inhibit the expression of cGAS and STING, while the expression level of NCOA4 was also suppressed. Notably, immunofluorescence staining showed that LOX-1 silence reduced the co-localization of STING and NCOA4 in HUVECs (Fig. 9C), suggesting that the mechanism of LOX-1-induced ferroptosis in endothelial cells may be related to the activation of the cGAS-STING pathway to regulate the expression of NCOA4.

To further confirm the effect of the cGAS-STING pathway on the regulatory mechanism of NCOA4 in ox-LDL-induced HUVECs, HUVECs were treated with the cGAS-STING pathway inhibitor RU.521 and measured cell viability. As shown in Supplementary Fig. S9A, RU.521 significantly suppressed ox-LDL-induced loss of cell viability. RU.521 significantly inhibited the release of inflammatory factors compared with the ox-LDL group (Supplementary Figs. S9B–D), while NCOA4 overexpression reversed the alleviating effect of RU.521, suggesting that the cGAS-STING pathway induces HUVECs inflammatory injury by up-regulating NCOA4.

As shown in Fig. 9D–H, RU.521 reversed ox-LDL-induced SOD and GSH levels and inhibited the accumulation of ROS, MDA and total iron levels in HUVECs, while overexpression of NCOA4 re-increased iron and lipid peroxidation levels in HUVECs. As described above, RU.521 also significantly decreased the expression of TFR1 and ACSL4, and elevated GPX4 expression in HUVECs compared with ox-LDL group (Fig. 9I). Immunofluorescence assay showed that RU.521 increased ferritin levels in injured HUVECs, whereas overexpression of NCOA4 significantly reversed the increase in ferritin levels (Supplementary Fig. S10A). Furthermore, the expression of ferritinophagy-related proteins was also affected, LC3II/Ⅰ and NCOA4 expression was significantly up-regulated, while P62 and ferritin expression was reduced in ox-LDL-induced HUVECs after RU.521 treatment (Supplementary Fig. S10B). Combined, our findings propose that cGAS- STING signaling pathway is activated by LOX-1 and up-regulates NCOA4 expression, activating ferritinophagy to promote ox-LDL-induced HUVECs ferroptosis.

### 3.10 Inhibiting the cGAS-STING pathway reduces NCOA4 nuclear translocation and STING-NCOA4 binding in HUVECs

**Fig. 10:**
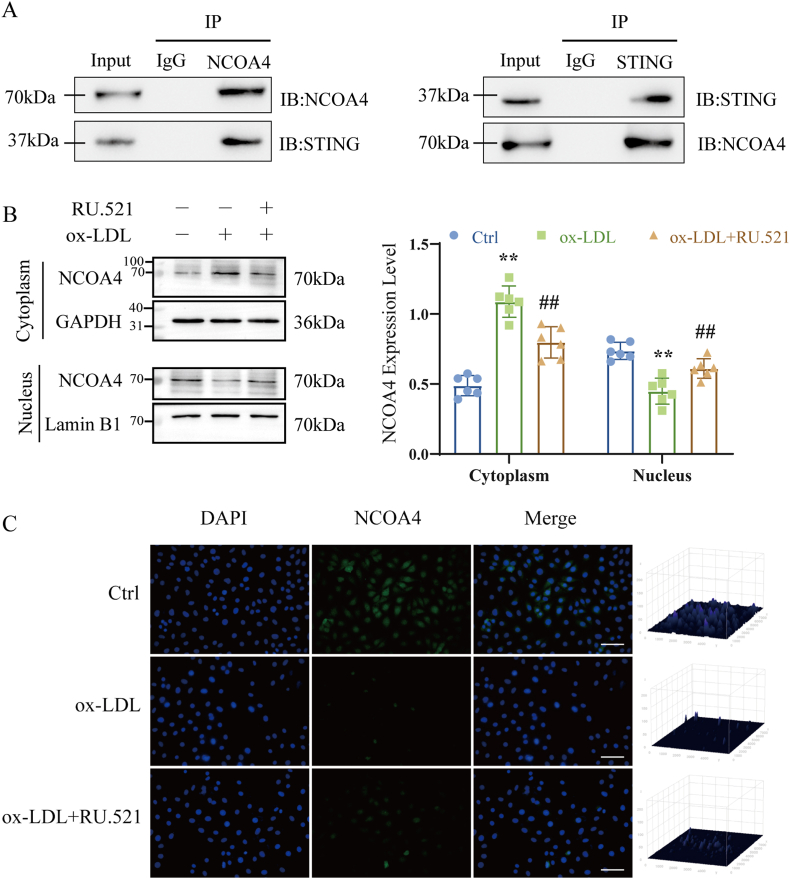
Inhibition of cGAS-STING pathway reduces NCOA4 nuclear translocation in HUVECs. (A) Co-IP to validate the interaction between STING and NCOA4. (B) Western blot to detect NCOA4 expression in cytoplasm and nucleus (n = 6). (C) Immunofluorescence assay to detect fluorescence of NCOA4 in the nucleus (scale bar: 100 μm, n = 3). ∗*P* < 0.05, ∗∗*P* < 0.01 vs. control group, ^#^*P* < 0.05, ^##^*P* < 0.01 vs. ox-LDL group.

To elucidate the role of the cGAS-STING pathway in the transcriptional regulation of NCOA4 in injured HUVECs, we performed immunoprecipitation assays utilizing specific antibodies. STING co-precipitated with NCOA4 from the lysate of HUVECs under physiological conditions (Fig. 10A). Additionally, we observed a time-dependent downregulation of NCOA4 levels within the nucleus (Fig. 10B), suggesting a potential nuclear translocation of NCOA4. Immunofluorescence staining further revealed an increased nuclear translocation of NCOA4 in HUVECs following exposure to ox-LDL (Fig. 10C). Taken together, these results confirm that the cGAS-STING pathway enhances the interaction of STING with NCOA4, thereby facilitating the role of NCOA4 nuclear translocation.

### 3.11 A key target of GLXB anti-AS strategy is inhibition of the LOX-1 receptor

GLXB has a rich history of use in the treatment of AS-related diseases, and our research group has demonstrated its significant anti-atherosclerotic effects [29]. The therapeutic effects of drug components primarily involve triggering biological responses in cells by binding to specific targets. In our previous study, it was discovered that GLXB can prevent endothelial injury in AS by inhibiting ferroptosis [30], but the exact targets and interactions of the drug were not clear. However, the current study has identified LOX-1 as a crucial target for intervention in the AS disease. Therefore, further investigation is needed to determine whether LOX-1 is an important target for GLXB therapy in regulating ferroptosis.

**Fig. 11:**
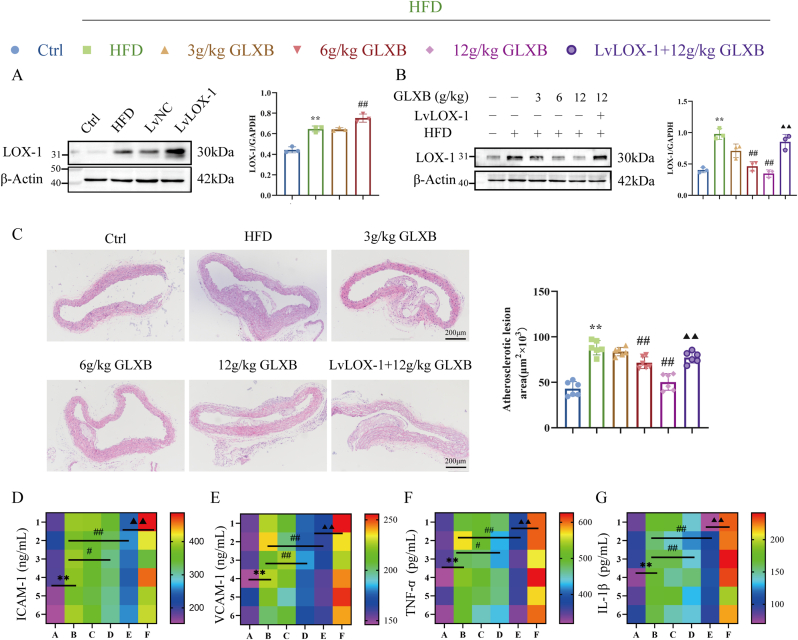
Effect of GLXB on atherosclerotic through LOX-1 receptor. (A–B) Western blot to detect the infection efficiency of injected overexpressed LOX-1 lentivirus and the expression of LOX-1 protein by GLXB (n = 3). (C) HE staining to observe the aortic plaque area (n = 6). (D–G) Measurement of inflammatory factors levels were determined within the aorta using commercially respective kits (n = 6). ∗*P* < 0.05, ∗∗*P* < 0.01 vs. control group; ^#^*P* < 0.05, ^##^*P* < 0.01 vs. HDF group; ^▲^*P* < 0.05, ^▲▲^*P* < 0.01 vs. 12g/kgGLXB group.

To explore this, we employed an ApoE^−/−^ mice model overexpressing LOX-1 and assessed the efficacy of overexpression as well as the impact of GLXB on aortic LOX-1 protein expression using western blot analysis. The findings revealed that GLXB effectively reversed the HFD-induced upregulation of protein expression in a dose-dependent manner (Fig. 11A–B), indicating that the LOX-1 receptor is indeed a key target of GLXB. Furthermore, treatment with GLXB significantly reduced aortic arch plaque area and intima-media thickness compared to the HDF group; however, overexpression of LOX-1 reversed this therapeutic effect on AS (Fig. 11C). To further explore the influence of GLXB on aortic inflammatory injury in AS mice mediated by LOX-1, we conducted an analysis of the release of inflammatory factors in the aorta of AS mice. As shown in Fig. 11D–G, it was observed that GLXB significantly elevated the level of inflammatory factor release; however, this effect was notably reversed by overexpression of LOX-1. These findings suggest that GLXB mitigated HFD-induced AS by inhibiting the LOX-1 receptor.

### 3.12 GLXB exerts anti-AS effects by preventing NCOA4-mediated aortic ferroptosis via LOX-1 inhibition of the cGAS-STING pathway

**Fig. 12:**
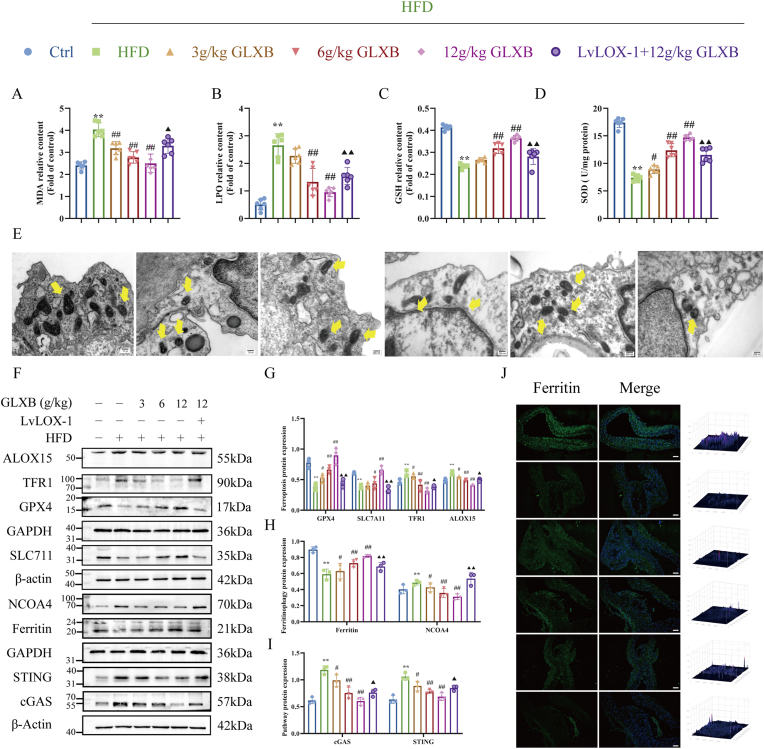
GLXB prevents NCOA4-mediated aortic ferroptosis and exerts anti-AS effects by inhibiting LOX-1 in the cGAS-STING pathway. (A–D) Biochemical kit to detect aortic lipid peroxidation levels (n = 6). (E) Transmission electron microscopy to detect mitochondrial morphological damage in aortic endothelial cells (n = 3). (F–H) Western blot to detect aortic ferroptosis protein levels, ferritinophagy protein level and cGAS-STING pathway protein level (n = 3). (I) Immunofluorescence observation of ferritin expression in aorta (n = 3). ∗*P* < 0.05, ∗∗*P* < 0.01 vs. control group, ^#^*P* < 0.05, ^##^*P* < 0.01 vs. HDF group; ^▲^*P* < 0.05, ^▲▲^*P* < 0.01 vs. 12 g/kg GLXB group.

To investigate whether the anti-atherosclerotic effect of GLXB by inhibiting ferroptosis was related to the regulation of LOX-1, our research team detected levels of ferroptosis in the aortic. As illustrated in Fig. 12A–D, GLXB can reduce the excessive accumulation of MDA and LPO, and increase the expression levels of GSH and SOD in the aorta of AS mice, whereas overexpression of LOX-1 reversed the inhibitory effect of GLXB on HFD-induced lipid peroxide levels. Furthermore, TEM revealed that GLXB intervention significantly alleviated mitochondrial damage and the mitochondrial structure was basically restored compared with the HFD group, while overexpression of LOX-1 significantly aggravated the mitochondrial damage and reversed the therapeutic effect of GLXB (Fig. 12E). Consistent with this result, GLXB significantly increased the protein expression of GPX4 and SLC7A11, and inhibited the expression of TFR1 and ALOX15, whereas overexpression of LOX-1 reversed the therapeutic effect of GLXB (Fig. 12F and G), indicating that GLXB could inhibits ferroptosis via down-regulating LOX-1 in AS mice.

To investigate the role of GLXB in regulating LOX-1 and its relationship with ferroptosis through ferritinophagy, our research team assessed the levels of ferritinophagy in the aorta. Compared with the HFD group, treatment with GLXB significantly downregulated the expression level of NCOA4 protein while concurrently increasing ferritin protein expression in a dose-dependent manner. Moreover, fluorescence microscopy results for ferritin corroborated these findings, confirming that GLXB treatment markedly suppressed ferritinophagy levels in the aorta of atherosclerotic mice by inhibiting LOX-1. As expected, the protein expression levels of cGAS and STING in the aorta of mice treated with GLXB were significantly decreased compared to the HFD group. The overexpression of LOX-1 was able to effectively reverse the impact of GLXB (Fig. 12F–I). In summary, these findings demonstrate that GLXB exerts anti-atherosclerotic effects by inhibiting NCOA4-mediated ferroptosis in the aorta via LOX-1 inhibition, which subsequently suppresses activation of the cGAS-STING pathway.

## 4 Discussion

**Fig. 13:**
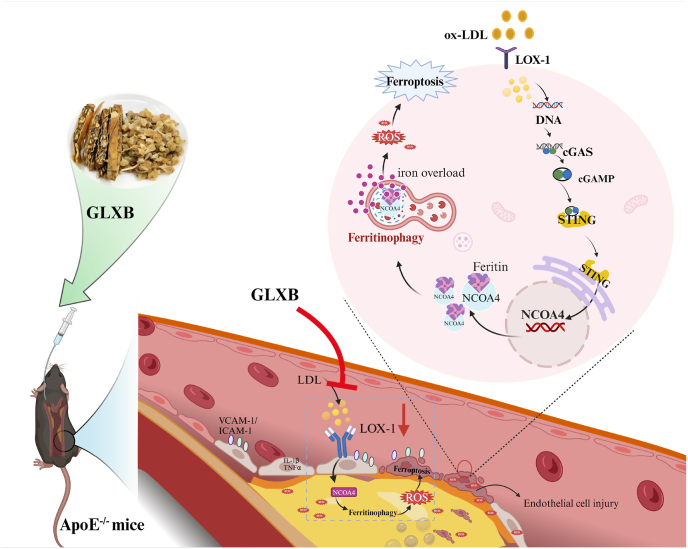
Schematic representation of the mechanism of action of NCOA4-mediated ferritinophagy-induced ferroptosis promoting endothelial injure. NCOA4-mediated ferritinophagy-induced ferroptosis is a key mechanism promoting endothelial injury. The key upstream regulator of NCOA4 expression is associated with cGAS-STING, which is a downstream signaling pathway activated by the LOX-1 receptor.

AS is a chronic vascular disease characterised by inflammation and lipid deposition in the arterial wall caused by endothelial injury [30,31]. Various pathological mechanisms contribute to the progression of AS [2]. Among these mechanisms, ferroptosis plays an important role in promoting AS development [8,9]. Our recent data indicate that ferroptosis may promote endothelial injury and plaque formation [30]. However, the underlying mechanism by which ferroptosis-mediated cell death contributes to AS remains unclear. In this study, we have discovered that NCOA4 is upregulated in AS and serves as a critical upstream regulator of ferroptosis. Mechanistically, LOX-1 activation enhances the expression of the ferritinophagy receptor NCOA4 via the cGAS-STING pathway, promoting STING-NCOA4 interactions to drives ferritinophagy-mediated iron mobilization and exacerbates lipid peroxidation. Notably, GLXB as a potent anti-atherosclerotic herbal compound, can modulate LOX-1 to inhibit cGAS-STING signaling pathway activation and down-regulate NCOA4-mediated ferritinophagy-induced ferroptosis in endothelial cells (Fig. 13).

As a chronic inflammatory disease and the initiation of AS pathogenesis is associated with vascular endothelial injury [32]. The release of inflammatory factors and the accumulation of lipids are key to the formation of vascular endothelial injury [33]. It is well established that intercellular adhesion molecules such as ICAM-1 and VCAM-1 are significantly expressed during AS, facilitating the adhesion of various inflammatory mediators, including TNF-α and IL-1β, which contribute to endothelial cell injury through multiple mechanisms [34,35]. Previous studies have shown that Nrf2-mediated ferroptosis is a causative factor in inflammatory diseases and AS [30]. Consistently, we found NCOA4 deficiency reduced the release of inflammatory factors and inhibited AS plaque formation. The use of ox-LDL-stimulated HUVECs to mimic vascular endothelial cell injury in vitro has also been demonstrated [30,36]. In ox-LDL-stimulated injured endothelial cells, cell viability was decreased and the release of inflammatory factors was increased. Conversely, silencing NCOA4 effectively ameliorated the release of inflammation-related factors and preserved endothelial function. Our results provide evidence that NCOA4 is involved in the process of atherosclerotic endothelial injury.

Herein, we demonstrated that NCOA4-mediated ferroptosis represents a novel mechanism underlying ox-LDL-induced inflammatory injury in endothelial cells. Ferroptosis is a newly identified form of regulated cell death characterized by mitochondrial morphological damage, the release of iron ions, and the degradation of antioxidant enzymes or ferritin [37]. Our findings reveal that both NCOA4 knockout and silencing effectively inhibit ferroptosis in vitro and in vivo, while overexpression of NCOA4 further enhances indicators of ferroptosis. Consistently, we observed an increase in lipid peroxidation and elevated iron levels (two classic marker of ferroptosis) in HUVECs treated with ox-LDL. GSH is a crucial antioxidant and free radical scavenger in the body, regulated at the transcriptional level by the NFE2L2/NRF2 signaling pathway [8]. We hypothesize that the potential reasons for the elevated GSH expression levels observed upon NCOA4 knockout treatment in cells include: First, inhibiting ferroptosis contributing to reduced GSH depletion; furthermore, this observation could be linked to the activation of NRF2-dependent transcriptional pathways. Additionally, we found that Ferristatin-1 (an inhibitor of erastin-induced ferroptosis) significantly alleviated ox-LDL-induced cell death in HUVECs, whereas overexpression of NCOA4 markedly reversed this effect. These results indicate that ferroptosis plays a crucial role in ox-LDL-induced endothelial cell death and highlight NCOA4 as a key mediator of this process.

Ferritinophagy is a selective autophagy responsible for intracellular ferritin degradation and is mainly mediated by the cargo receptor NCOA4 [38]. Recent studies have shown that NCOA4-associated ferritinophagy plays an important role in ferroptosis and in different cell types [39], suggesting that NCOA4-mediated ferritinophagy may be involved in the ferroptotic process within atherosclerotic endothelial cells. Our findings indicate that overexpression of NCOA4 further increased Ferritin and LAMP2 co-localization in the aortas of AS mice, whereas Ferritin protein expression levels were significantly reduced. Furthermore, the expression of ferritin was found to be upregulated in NCOA4-silenced HUVECs, while the co-localization of LC3 with ferritin expression was diminished. This suggests that NCOA4-mediated ferritin phagocytosis plays a central role in endothelial cell ferritinophagy. However, further research is necessary to explore the relationship between ferritinophagy and ferroptosis. To this end, we added the classical autophagy inhibitor (3-MA), which significantly inhibited the ferroptosis levels and increased the cell viability, suggesting that ferroptosis is regulated by autophagy and that inhibition of autophagy can reduce ferroptosis.

Subsequent studies have thus concentrated on the role of NCOA4-mediated ferritinophagy in endothelial cell ferroptosis, as well as the key upstream factors that regulate this process. In endothelial cells, several major scavenger receptors are critical for the active uptake of modified lipoproteins, including CD36, SR-A, and LOX-1 [24]. Previous studies have shown that LOX-1 is highly expressed in the vessel wall during the induction of atherosclerotic lesion formation [40]. In this study, we found that LOX-1 was significantly upregulated in aortic endothelial cells and injured HUVECs. Furthermore, silencing LOX-1 enhanced cell viability while reducing levels of inflammatory factors and ferroptosis markers, thereby contributing to the inhibition of AS.

The cGAS-STING signaling pathway is an important DNA sensing mechanism that mediates the innate immune response [41]. The results of the comet assay conducted in this study indicated that ox-LDL increased the tailing phenomenon in HUVECs, with a time-dependent escalation in the degree of tailing, suggesting an increase in DNA damage in injured HUVECs. Previous studies have shown that activation of the cGAS-STING pathway plays a pivotal role in aging-related endothelial dysfunction. Notably, inhibition or silencing of cGAS or STING in human aortic endothelial cells significantly enhances endothelial function and reverses elevated expression levels of aging markers [42,43]. Our study further revealed that the cGAS-STING inhibitor RU.521 effectively inhibited the release of inflammatory factors and significantly reversed ox-LDL-induced HUVECs injury. Additionally, several studies have shown that the cGAS-STING pathway is closely related to autophagy, highlighting its critical role in inducing ferroptosis [44]. In hepatic stellate cells, mitochondrial or genomic DNA stress drives in pancreatic cancer cells, mitochondrial or genomic DNA stress drives STING activation, which induces ferroptosis in an autophagy-dependent manner [45]. Recent studies have reported that the cGAS-STING pathway may exacerbate cerebral ischaemia-reperfusion injury by modulating NCOA4-mediated ferritinophagy [46]. However, the involvement of the cGAS-STING pathway in AS through the regulation of ferritinophagy remains unexplored. In this experiment, we demonstrated that silencing LOX-1 significantly inhibit the expression levels of cGAS and STING proteins. Interestingly, we observed a concurrent reduction in NCOA4 expression levels, which aligns with our findings that LOX-1 silencing markedly decreased the co-localization of STING and NCOA4. To further investigate the relationship between STING and NCOA4 in endothelial injury pathogenesis, we employed nuclear localization fluorescence and immunoprecipitation techniques. The application of the STING pathway inhibitor RI.521 resulted in increased nuclear localization of NCOA4 while simultaneously reducing its interaction with STING. These results suggest that LOX-1 may regulate NCOA4 expression through activation of the cGAS-STING signaling pathway.

GLXB is a classic combination of medications used to treat thoracic paralysis syndrome caused by phlegm and stagnant stagnation, as described in the Essentials of the Jin gui Yao lue [29]. Modern medicine has demonstrated that GLXB co-administration is mainly used for lowering serum lipids, reducing aortic plaque and treating AS-related diseases [30]. In this study, we conducted an investigation into the preventive effect of GLXB on AS in ApoE^−/−^ mice. The findings revealed that GLXB has the ability to inhibit the formation of AS plaques and improve the narrowing of vascular lumen. Additionally, it significantly reduced the level of inflammatory factors in the aorta of atherosclerotic mice. In our previous study, we discovered that GLXB downregulated the expression of TFR1 and ALOX15 proteins while up-regulating the expression levels of negatively regulated ferroptosis -related proteins GPX4 and SLC7A11 in the aorta of atherosclerotic mice, reduced protein accumulation, decreased the level of lipid peroxidation in aortic tissue, and inhibited mitochondrial damage in aortic endothelial cells. Importantly, we found that tail vein injection of over-expressed lentiviral LOX-1 reversed the therapeutic effect of GLXB. Interestingly, we found that GLXB was able to dose-dependently inhibit NCOA4 protein expression and significantly increase ferritin protein expression levels; GLXB was also able to affect the cGAS-STING pathway, whereas overexpression of LOX-1 was able to inhibit the effects of GLXB. These results tentatively suggest that GLXB can down-regulate NCOA4 expression and reduce ferritinophagy-induced ferroptosis by inhibiting LOX-1 receptor activation and regulating the downstream cGAS-STING pathway, providing a molecular mechanism for us to subsequently investigate the inhibition of aortic endothelial injury to play a role in resisting the progression of AS.

In summary, we have shown that NCOA4-mediated ferritinophagy induces ferroptosis in endothelial cells and that the molecular mechanism controlling NCOA4 expression involves activation of the LOX-1-mediated cGAS-STING pathway and our findings provide new insights into the progression and treatment of AS.

## CRediT authorship contribution statement

**Li Zhu:** Writing – review & editing, Writing – original draft, Investigation. **Zijian Liu:** Formal analysis. **Jiahui Liu:** Formal analysis. **Zhenglong Li:** Validation, Methodology. **Youli Bao:** Formal analysis. **Xin Sun:** Visualization. **Wenchen Zhao:** Writing – review & editing. **An Zhou:** Writing – review & editing. **Hongfei Wu:** Writing – original draft, Supervision.

## Declaration of competing interest

All authors declare that they have no conflict of interest.

## Data Availability

The data that has been used is confidential.
