# Supplementary material for: NCOA4 linked to endothelial cell ferritinophagy and ferroptosis:a key regulator aggravate aortic endothelial inflammation and atherosclerosis
Source: Redox Biol. 2024 Dec 12;79:103465. doi: 10.1016/j.redox.2024.103465 (PMC11729014; doi:10.1016/j.redox.2024.103465)
Supplement: Multimedia component 1 [file mmc1.docx]

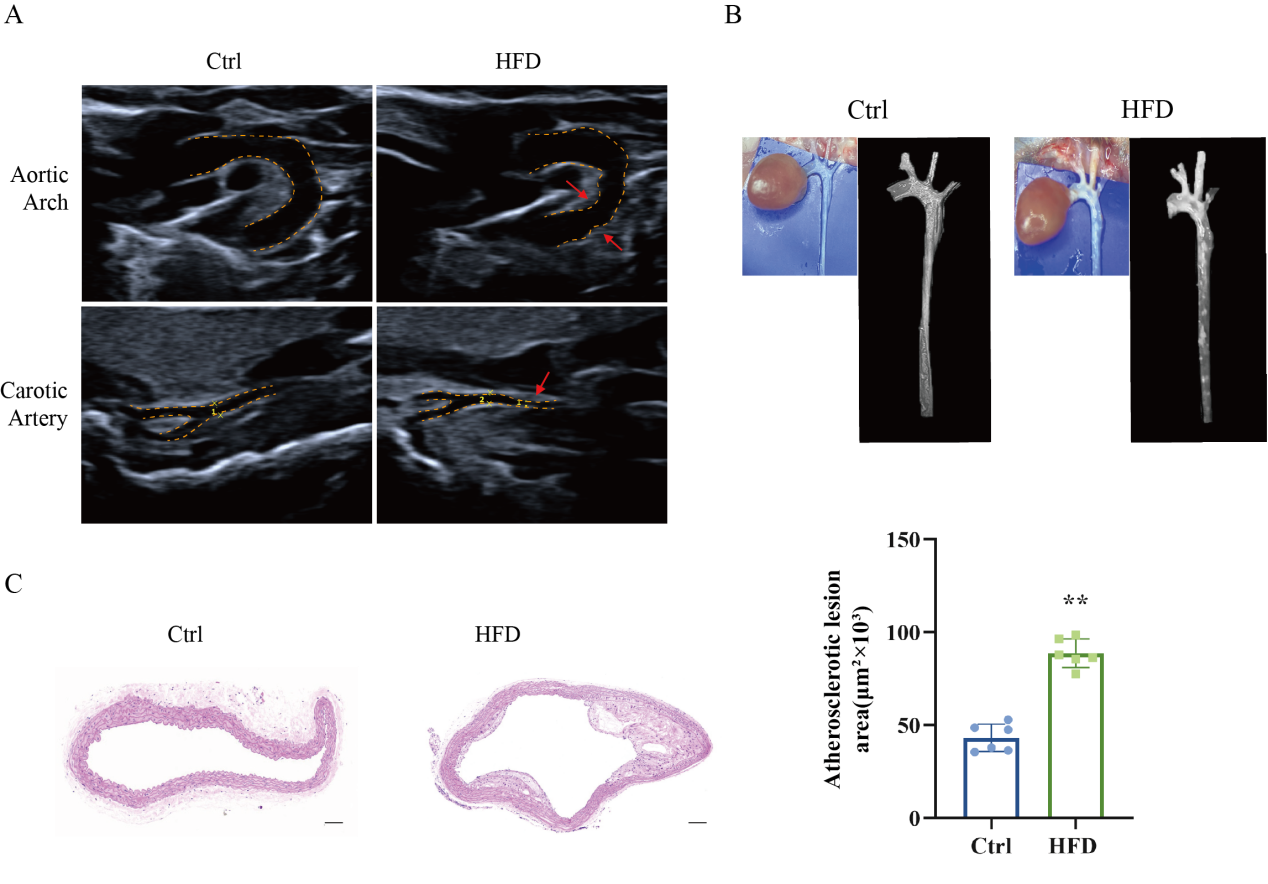


**Supplementary Fig. 1. The model of atherosclerosis was established in HFD-fed ApoE^-/-^ mice.** (A) An ultrasound imaging system was used to record the vascular wall thickness and plaque formation in the aortic arch and carotic artery (n=3). (B) Representative images of a normal aorta and atherosclerotic aorta (n=3). (C) HE staining of aortic arch (scale bar: 200µm, n=3). ***P*< 0.01 vs. control group.


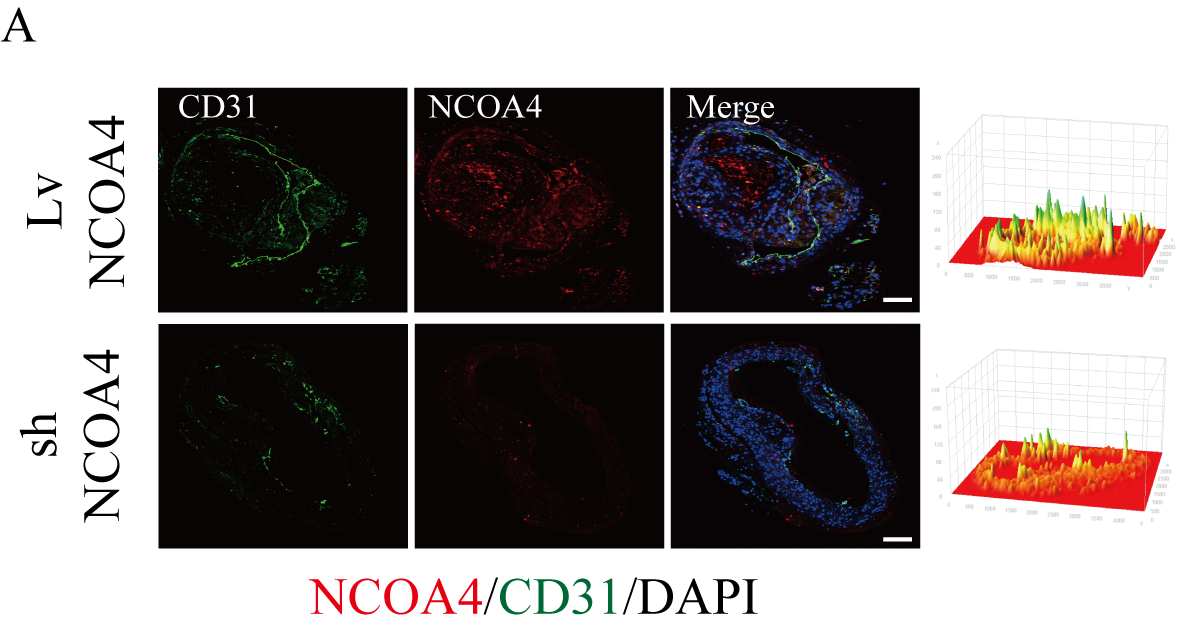


**Supplementary Fig. 2. Effects of shNCOA4 or LvNCOA4 on NCOA4 expression in endothelial cells of ApoE^-/-^ mice.** (A) Immunofluorescence staining of CD31 (green), NCOA4 (red) in mouse aorta (scale bar: 200 µm, n=3).


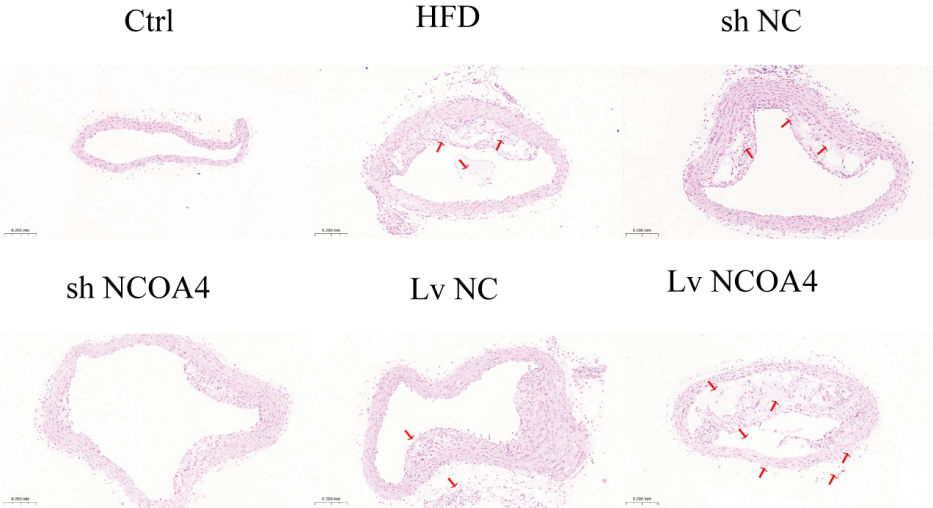


**Supplementary Fig. 3.** Prussian blue staining of aortic in each group (scale bar: 200µm, n=3).


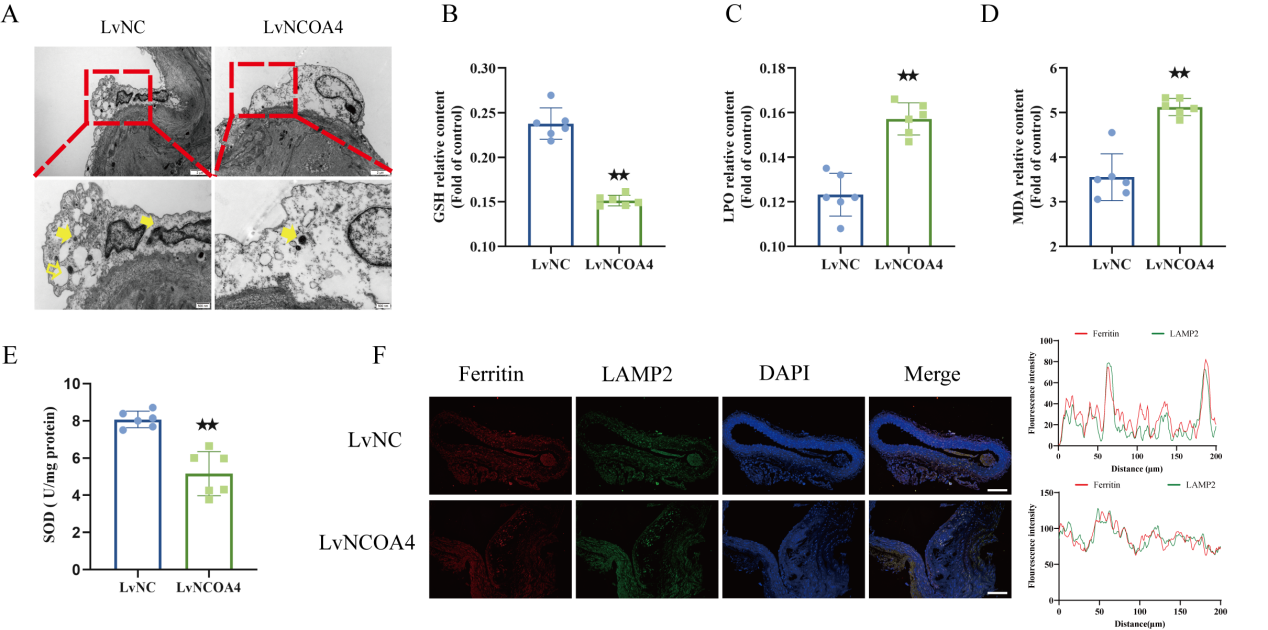


**Supplemental Fig. 4. Overexpression of NCOA4 promotes ferroptosis and the level of ferritinophagy in atherosclerotic endothelial cells.** (A) TEM to detect the mitochondrial injury in the aortic endothelial cells of AS mice (n=3). (B-E) Biochemical kits to detect the expression of lipid peroxidation levels of MDA, LPO, GSH, and SOD in the aortic tissues of AS mice (n=6). (F) Immunofluorescence staining of Ferritin (red) LAMP2 (green) are co-localized in sections of atherosclerotic lesions (scale bar: 100µm, n=3). ^★^*P* < 0.05, ^★★^P < 0.01 vs. LvNC group.


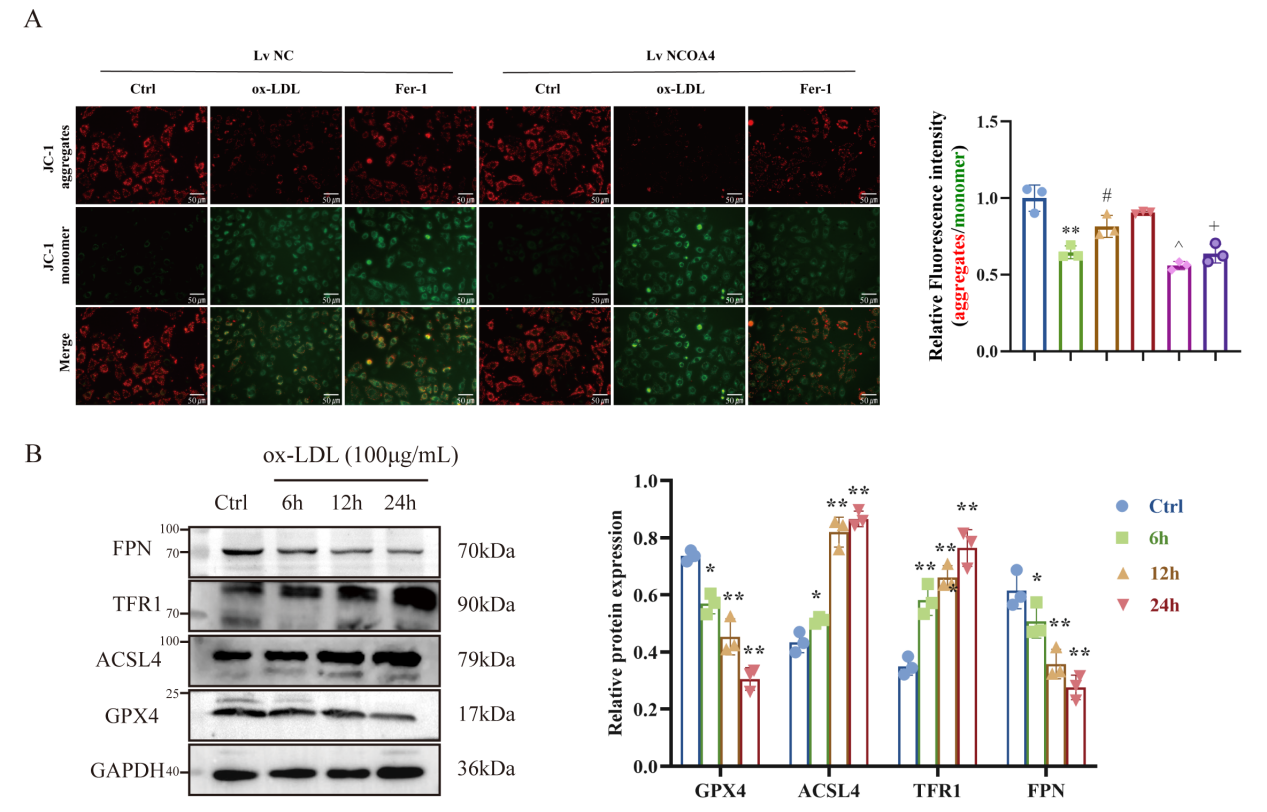


**Supplemental Fig. 5. NCOA4 overexpression aggravated ferroptosis in HUVECs induced by ox-LDL.** (A) Immunofluorescence to detect intracellular Mitochondrial membrane potential (n=3). (B) Western blot to detect the expression levels of GPX4, ACSL4, TFR1 and FPN proteins in HUVECs treated with ox-LDL for 6, 12, 24h (n=3). **P* < 0.05, ***P* < 0.01 vs. control group, ^#^*P* < 0.05 vs LvNC+ox-LDL, ^^^*P* < 0.05 vs LvNCOA4 group, ^+^*P* < 0.05 vs LvNCOA4+ox-LDL group.


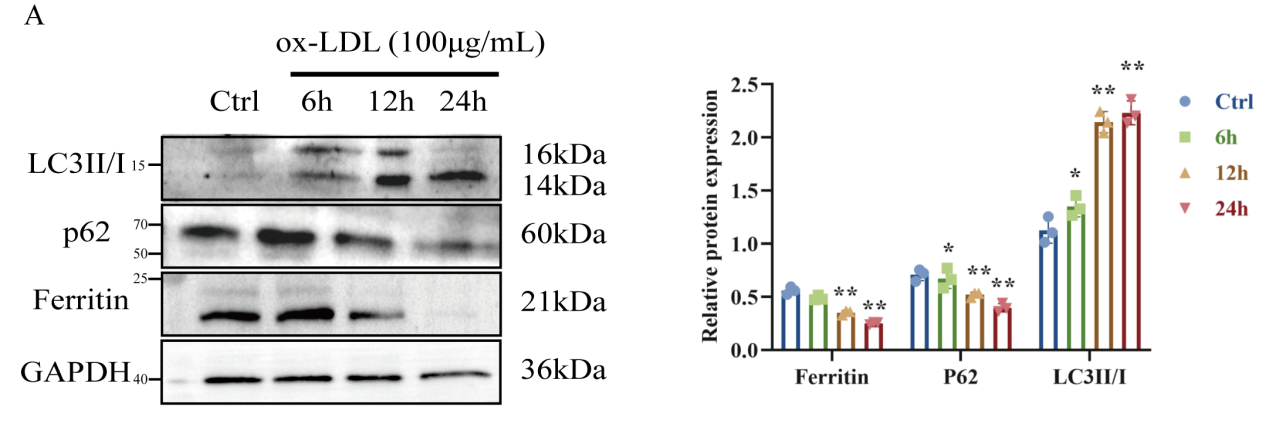


**Supplemental Fig. 6. The ferritinophagy in HUVECs was induced by ox-LDL.** Western blot to detect the expression levels of LC3Ⅱ/Ⅰ, P62 and Ferritin proteins in HUVECs treated with ox-LDL for 6, 12, 24h (n=3). **P* < 0.05, ***P* < 0.01 vs. control group.


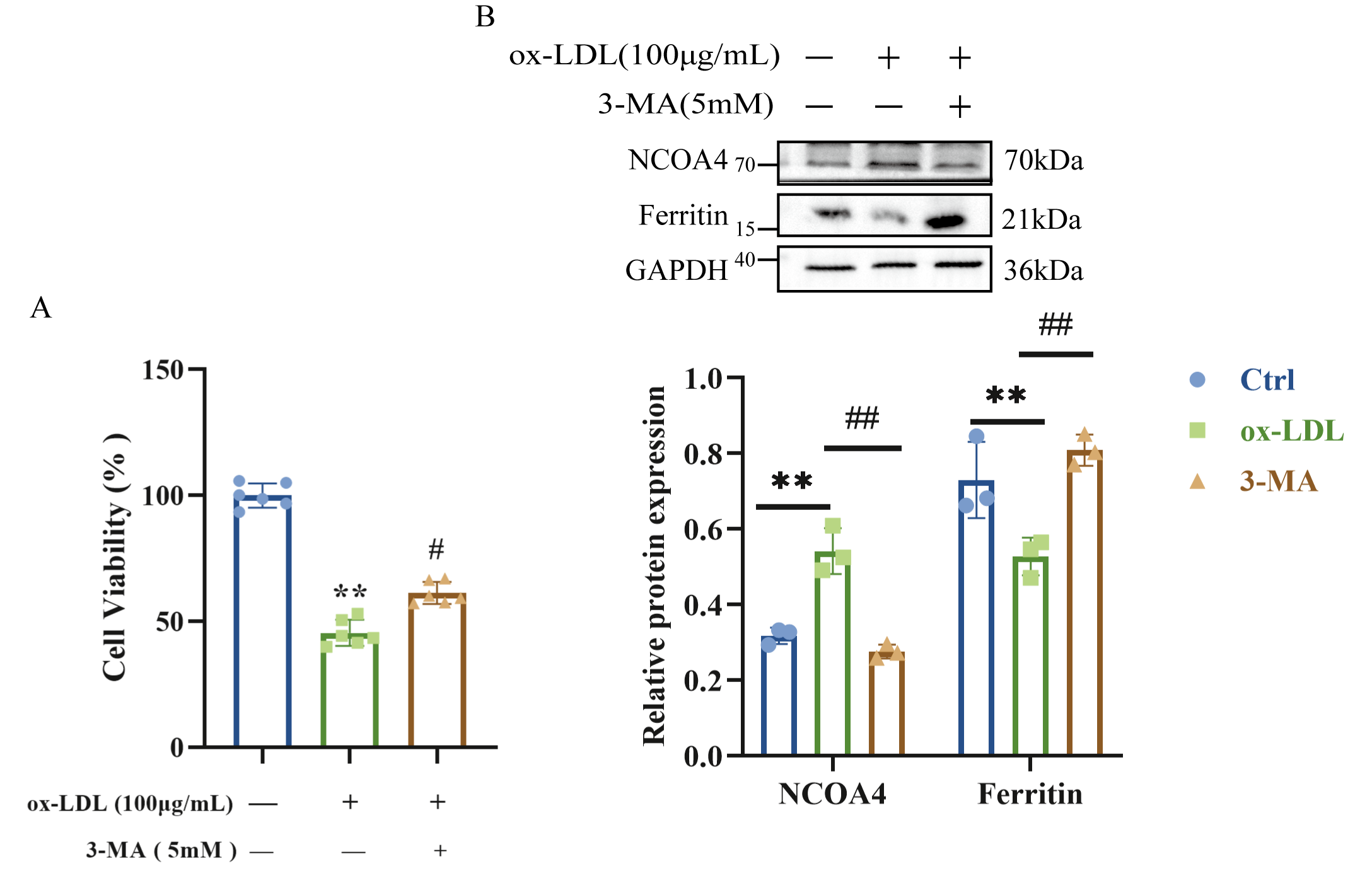


**Supplementary Fig. 7. 3-MA improves cell viability of ox-LDL treated HUVECs.**

HUVECs were pretreated with 3-MA (5mM) for 1h and then treated with ox-LDL (100μg/mL) for 24 h. (A) Cell viability was analysed by CCK-8 assay (n=6). (B) Western blot detect the levels of ferritinophagy-associated protein NCOA4 and Ferritin. **P* < 0.05, ***P* < 0.01 vs. control group, ^#^*P* < 0.05, ^##^*P* < 0.01 vs. ox-LDL group.


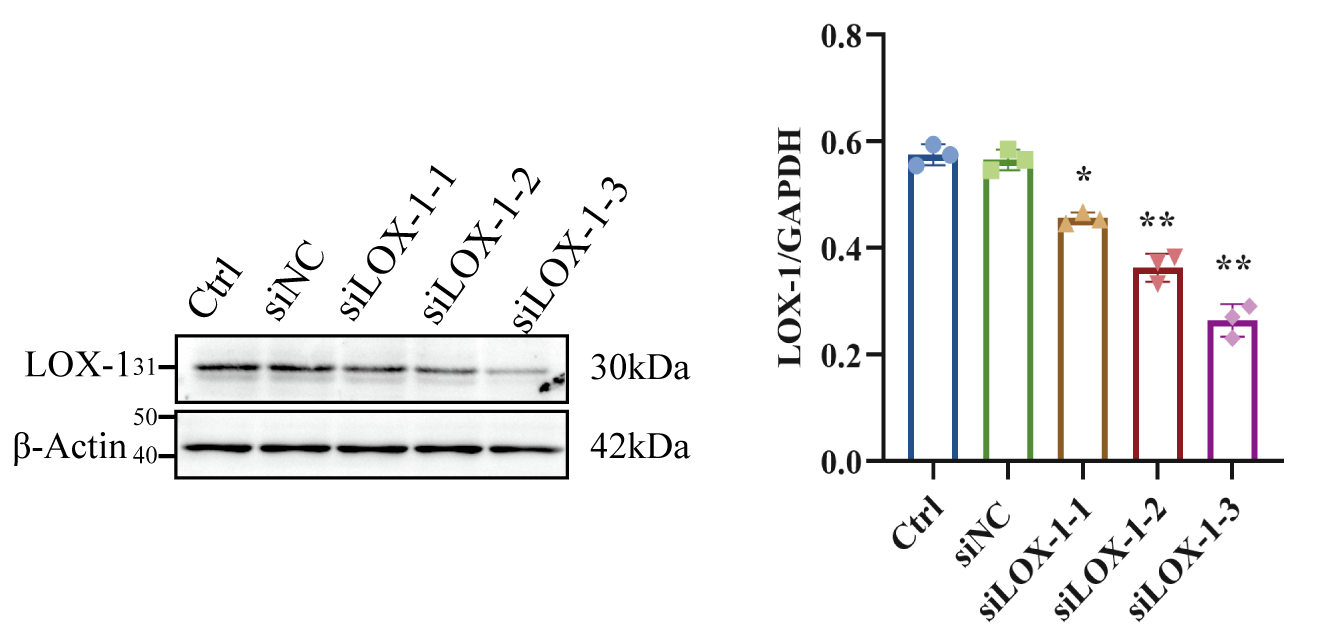


**Supplementary Fig. 8. Silencing LOX-1 in HUVECs.** Western blot to detect the transfection efficiency of silencing LOX-1 in HUVECs (n=6). **P* < 0.05, ***P* < 0.01 vs. control group.


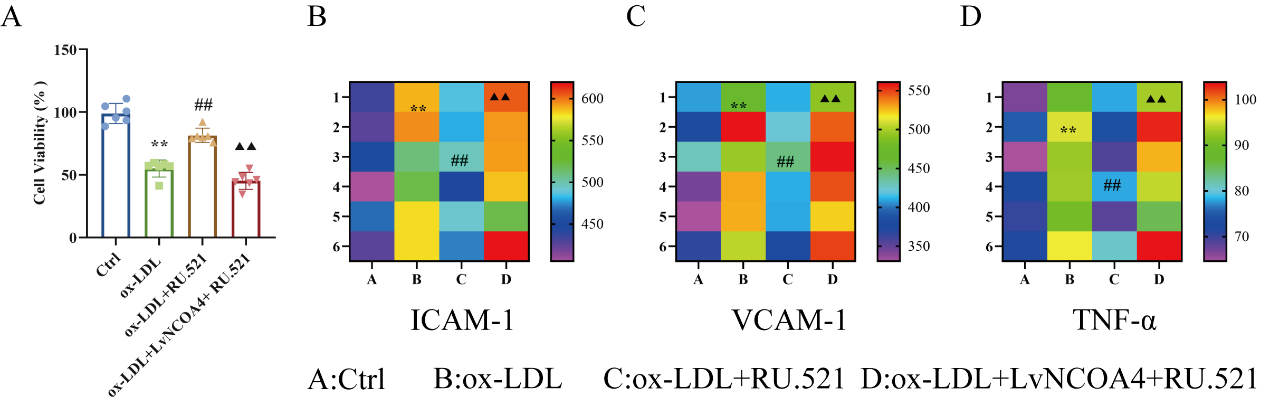


**Supplementary Fig. 9. RU.521 alleviates cellular inflammatory injury in ox-LDL-treated HUVECs.** HUVECs were pretreated with RU.521 (10μmol/mL) for 1 hour, then treated with ox-LDL (100μg/mL) for 24 hours. (A) Cell viability was analysed by CCK-8 assay, n=6. (B-D) ELISA kit to detect ICAM-1, VCAM-1 and TNF-α levels, n=6. **P* < 0.05, ***P* < 0.01 vs. control group, ^#^*P* < 0.05, ^##^*P* < 0.01 vs. ox-LDL group. ^▲^*P* < 0.05, ^▲▲^*P* < 0.01 vs. ox-LDL+RU.521 group.


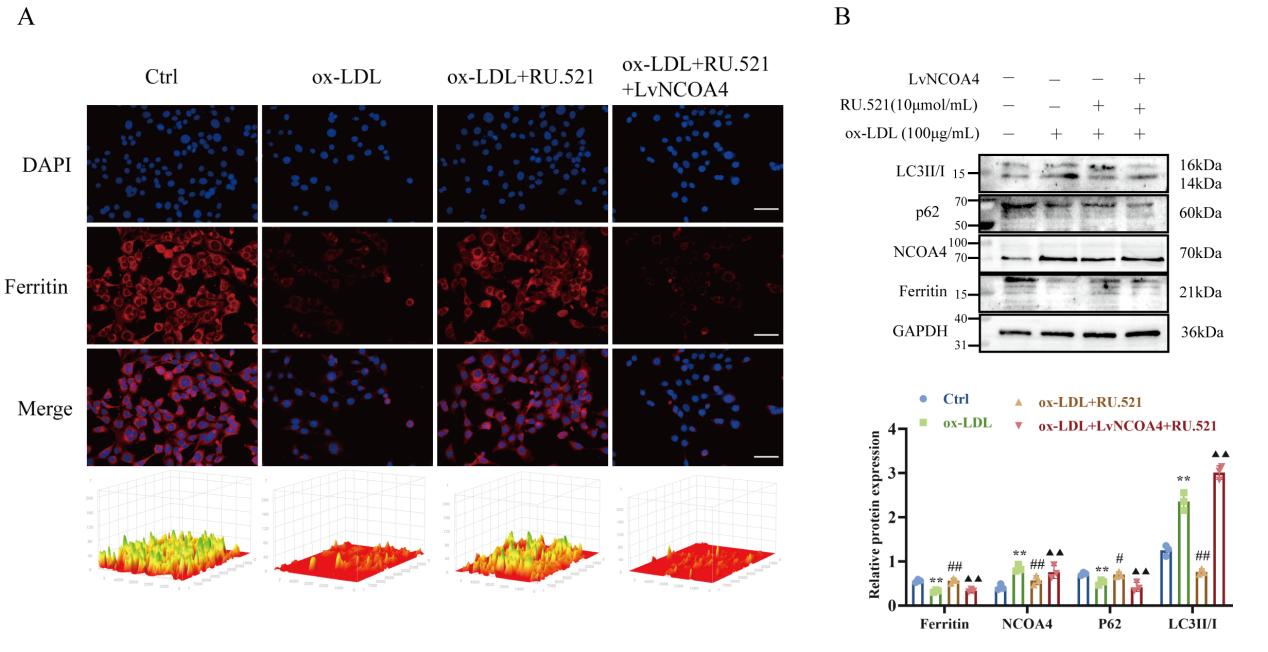


**Supplementary Fig. 10. RU.521 alleviates cellular ferritinophagy in ox-LDL-treated HUVECs.** (A) Immunofluorescence to detect the levels of ferritin in HUVECs. (scale bar: 100µm, n=3). (B) Western blot to detect ferritinophagy protein (n=3). **P* < 0.05, ***P* < 0.01 vs. control group, ^#^*P* < 0.05, ^##^*P* < 0.01 vs. ox-LDL group. ^▲^*P* < 0.05, ^▲▲^*P* < 0.01 vs. ox-LDL+RU.521 group.

**Supplemental Table 1 The primer sequence**

| Primer | Sequence（5' to 3'） |
| --- | --- |
| LOX-1 | Forward Primer 5’-TTGCCTGGGATTAGTAGTGACC-3’  Reverse Primer 5’-GCTTGCTCTTGTGTTAGGAGGT-3’ |
| CD36 | Forward Primer 5’-CTTTGGCTTAATGAGACTGGGAC-3’  Reverse Primer 5’-GCAACAAACATCACCACACCA-3’ |
| SR-A | Forward Primer 5’-CCAGGTCCAATAGGTCCTCC-3’  Reverse Primer 5’-CTGGCCTTCCGGCATATCC-3’ |
| GAPDH | Forward Primer 5’-ACAACTTTGGTATCGTGGAAGG -3’  Reverse Primer 5’-GCCATCACGCCACAGTTTC-3’ |
